# Molecular bases for differential aging programs between flag and second leaves during grain-filling in rice

**DOI:** 10.1038/s41598-017-07035-9

**Published:** 2017-08-18

**Authors:** Shinyoung Lee, Hyobin Jeong, Sichul Lee, Jinwon Lee, Sun-Ji Kim, Ji-Won Park, Hye Ryun Woo, Pyung Ok Lim, Gynheung An, Hong Gil Nam, Daehee Hwang

**Affiliations:** 1 Center for Plant Ageing Research, IBS, Daegu Gyeongbuk Institute of Science and Technology, Daegu, 711-873 Republic of Korea; 2 Department of New Biology, Daegu Gyeongbuk Institute of Science and Technology, Daegu, 711-873 Republic of Korea; 3 Department of Plant Molecular Systems Biotechnology and Crop Biotech Institute, Kyung Hee University, Yongin, 446-701 Republic of Korea

## Abstract

Flag leaves (FL) and second leaves (SL) in rice show differential aging patterns during monocarpic senescence. Coordination of aging programs between FL and SL is important for grain yield and quality. However, the molecular bases for differential aging programs between FL and SL have not been systematically explored in rice. Here, we performed mRNA-sequencing of FL and SL at six time points during grain-filling and identified four molecular bases for differential aging programs between FL and SL: phenylpropanoid biosynthesis, photosynthesis, amino acid (AA) transport, and hormone response. Of them, photosynthesis (carbon assimilation) and AA transport (nitrogen remobilization) predominantly occurred in FL and SL, respectively, during grain-filling. Unlike other molecular bases, AA transport showed consistent differential expression patterns between FL and SL in independent samples. Moreover, long-distance AA transporters showed invariant differential expression patterns between FL and SL after panicle removal, which was consistent to invariant differential nitrogen contents between FL and SL after panicle removal. Therefore, our results suggest that the supplies of carbon and nitrogen to seeds is functionally segregated between FL and SL and that long-distance AA transport is an invariant core program for high nitrogen remobilization in SL.

## Introduction

In cereal crops, nutrients are predominantly transported to grains from leaves. In rice, leaves provide grains with carbon (C) and nitrogen (N) mainly through assimilation and remobilization, respectively^1–3^. Therefore, senescence of leaves greatly affects nutrient supply to grains^4–7^. During leaf senescence, photosynthesis (C assimilation) is down-regulated, whereas N remobilization is up-regulated^6, 7^. Thus, delayed senescence can result in increased grain yields by delaying down-regulation of C assimilation^8^. On the other hand, delayed senescence can decrease protein content (grain quality) in grains by delaying up-regulation of N remobilization, whereas accelerated senescence can increase protein content^9–11^. Collectively, these data indicate that leaf senescence is crucial for determining both yield and quality of grains during the grain-filling period.

To investigate leaf senescence at the molecular level in rice, several studies have identified a broad spectrum of genes that are associated with leaf senescence using genetic approaches. These include the following: (1) transcription factors (TFs), such as *OsWRKY42* ^12^, *OsDOS* ^13^, *OsTZF1* ^14^, *OsNAP* ^8^, and *OsNAC106* ^15^; (2) hormonal factors, such as the JA receptor CORONATINE INSENSITIVE 1b^16^; (3) defense-related proteins, such as lesion mimic and early senescence 1 (*OsLMES1*), spotted leaf3 (*OsSPL3*), and UDP-N-acetylglucosamine pyrophosphorylase 1 (*OsUAP1*)^17–19^; (4) phytochrome B (*OsPhyB*)^20^; and (5) the chlorophyll (Chl) degradation genes (*OsNYC1*, *OsPPH1*, *OsNYC4*, *OsRCCR1*, and *OsSGR1*)^21–26^. Moreover, time-course gene expression profiling of leaves during the grain-filling period can provide a more comprehensive understanding of leaf senescence. Recently, gene expression profiling of flag leaves (FL) from vegetative to senescence stages has identified senescence-associated genes (SAGs), including the six NAC TFs (*Os02g0579000*, *Os03g0327100*, *Os03g0327800*, *Os07g0566500*, *Os07g0683200*, and *Os10g0571600*)^27^.

These studies were performed mostly in FL only. However, senescence in rice does not occur independently in individual leaves and is a systemic process. To characterize this monocarpic senescence, Choudhuri’s *et al*.^28–32^ analyzed physiological changes, including transitions of Chl, protein, and RNA expression levels, in FL and second leaves (SL), which influence grain-filling the most^3, 33^. Comparisons of FL and SL showed that FL and SL have differential senescence patterns (i.e., rates of decreases in Chl and protein levels) during monocarpic senescence and that these differential senescence patterns were altered by panicle removal^28, 30^. However, FL and SL were shown to perform shared functions to provide C and N to grains^3, 33^. These data prompted us to characterize the shared and differential senescence programs in FL and SL and to determine the effects of grain-filling on the senescence programs of the two leaf types. Thus, we performed mRNA-sequencing of FL and SL at six time points during the grain-filling period and compared gene expression profiles between FL and SL. These analyses revealed SAGs with shared and differential patterns of expression changes during grain-filling. Mapping of these SAGs into cellular processes identified key cellular processes underlying the shared and differential senescence programs between FL and SL. Furthermore, among differential senescence programs, changes after panicle removal were used to distinguish invariable core senescence programs from variable senescence programs.

## Results

### Differential decreases in Chl levels and N contents between FL and SL during grain filling

**Figure 1:**
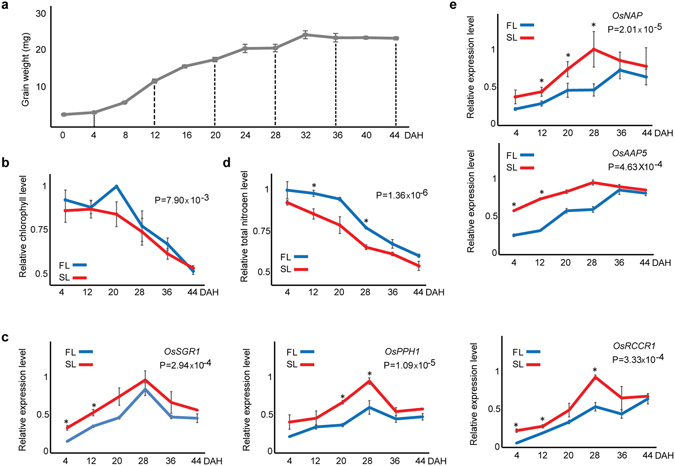
Differential decreases of Chl levels and N contents in FL and SL during the entire grain-filling period. (**a**) Temporal changes of average grain weights (mg) during grain-filling measured at 12 time points (4-day intervals from 0 DAH to 44 DAH). (**b**,**c**) Temporal changes of relative Chl levels (**b**) and expression levels of *OsSGR1*, *OsPPH1*, and *OsRCCR1* associated with Chl degradation (**c**) during grain-filling. (**d**,**e**) Temporal changes of relative total N content (**d**) and expression levels of *OsNAP* and *OsAAP5* associated with N remobilization (**e**) during grain-filling. Relative Chl levels and N content were determined by normalization to maximum levels in FL and SL during the grain-filling period. Relative gene expression levels were normalized to those of actin at corresponding time points. Data are presented as means ± SEM from three biological trials. P-values in (**b**–**e**) were computed using one-way ANOVA with post-hoc Bonferroni correction. *P < 0.05, two-tailed t-tests at each time point.

In preliminary analyses of the Dongjin (*Oryza sativa* L. ssp. japonica) variety, which has been widely used for genetic analyses^34^, differential patterns of Chl degradation were observed between FL and SL during grain-filling. Because this is a hallmark feature of leaf senescence, we used this variety for further experiments in this study. To systematically examine temporal characteristics of differential senescence patterns, we initially measured grain weights during the entire grain-filling period. Temporal grain weight patterns revealed that grain-filling was completed at around 32 days after heading (DAH) under our growth conditions (Fig. 1a). Accordingly, we selected six different time points (4, 12, 20, 28, 36, and 44 DAH) to cover the entire grain-filling period and examined Chl levels in FL and SL. Chl levels decreased during grain-filling and tended to be higher in FL than in SL (P < 0.01, two-way AVOVA with Bonferroni correction; Fig. 1b). To confirm this tendency, we measured mRNA expression levels of *OsSGR1*, *OsPPH1*, and *OsRCCR1*, which are reportedly associated with Chl degradation^22, 24–26^, and found higher levels in SL than in FL (P < 0.01, two-way AVOVA with Bonferroni correction; Fig. 1c), thus consistent with a higher Chl level in FL than in SL. Moreover, rice grains obtain N from leaves through N remobilization, which is another representative senescence feature^4, 35, 36^. Thus, we measured total N contents in FL and SL at the six time points. N contents decreased during grain-filling and were higher in FL than in SL (P < 0.01, two-way AVOVA with Bonferroni correction; Fig. 1d). To confirm this difference, we measured mRNA expression levels of *OsNAP* and *OsAAP5*, which are reportedly associated with N remobilization^8, 37^, and found higher levels in SL than in FL (P < 0.01, two-way AVOVA with Bonferroni correction; Fig. 1e), consistent with a higher N content in FL than in SL. However, expression levels of these genes decreased after peaking at 28 (*OsSGR1*, *OsPPH1*, and *OsRCCR1* in FL and SL; *OsNAP* and *OsAAP5* in SL) or 36 DAH (*OsNAP* and *OsAAP5* in FL), whereas Chl levels and N contents continuously decreased (see Discussion). Taken together, these data indicate that FL and SL have differential decreases in Chl levels and N contents, which are representative senescence phenotypes during the grain-filling period.

### SAGs with mRNA expression changes in FL and SL during grain-filling

**Figure 2:**
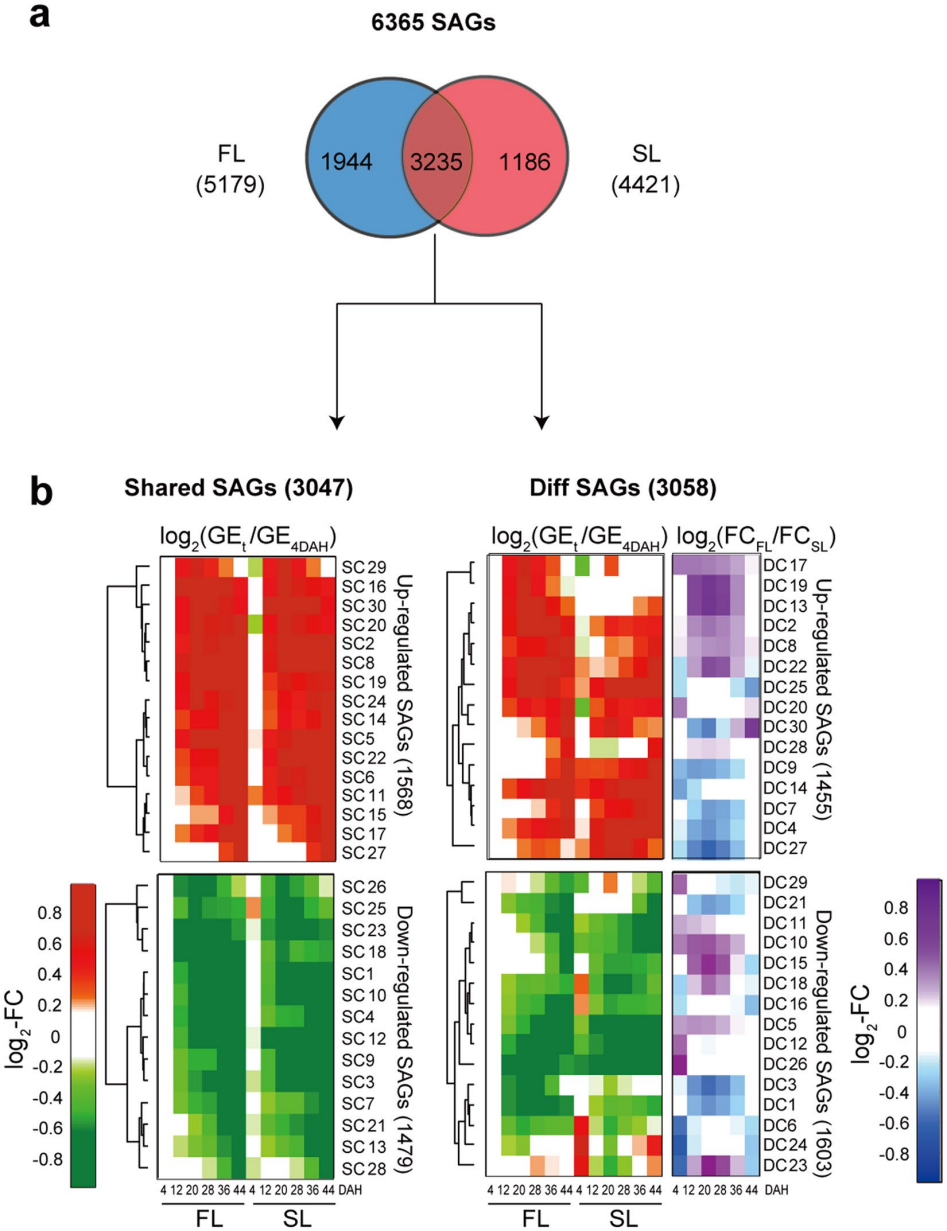
SAGs with shared and different expression patterns between FL and SL during grain-filling. (**a**) Relationships of SAGs with significant expression changes during grain-filling in FL and SL. (**b**) SAGs with shared (Shared_SAGs) and different (Diff_SAGs) temporal gene expression (GE) patterns between FL and SL. The 3047 Shared_SAGs were clustered into 30 clusters (SC1-30; Methods) that were divided into 16 up-regulated and 14 down-regulated clusters (top and bottom left heat maps, respectively). For the up- or down-regulated clusters, the heat map shows the average log_2_-fold-changes [log_2_-FC = log_2_(GE_*t*_/GE_4DAH_)] of genes at each time point (*t*) in FL and SL, compared with those at 4 DAH in FL. Up-regulation (red; log_2_-FC ≥0) and down-regulation (green; log_2_-FC < 0) in FL and SL during grain-filling are represented in comparison with those at 4 DAH in FL. The color bar shows the gradient of log_2_-FC at each time point compared with that at 4 DAH in FL. The 3058 Diff_SAGs were also clustered into 30 clusters (DC1-30), which were then divided into 15 up-regulated and 15 down-regulated clusters (top and bottom right heat maps, respectively). The same method was used to visualize the up- or down-regulated clusters of Diff_SAGs using the same color (red and green) gradient. Additional heat maps show differences in the average log_2_-FCs [log_2_(FC_FL_/FC_SL_)] values of genes in up- and down-regulated clusters between FL and SL at the six time points. Colors represent higher (purple) or lower (blue) log_2_-FCs in FL than in SL. The color bar shows the gradient of log_2_-fold-change differences between FL and SL at each time point.

To investigate the molecular nature of differential patterns of Chl levels and N contents between FL and SL during the grain-filling period, we performed mRNA-sequencing of FL and SL at the six time points used in the analyses described above. Subsequently, we used time-course mRNA-sequencing data and identified a total of 6365 SAGs that showed significant (P < 0.05, ANOVA with Bonferroni correction) mRNA expression changes in FL (5179 SAGs) and/or SL (4421 SAGs) during grain-filling (Methods; Supplementary Table 1). Comparisons of the SAGs in FL and SL showed that 3235 SAGs were common in both, whereas 1944 and 1186 SAGs were uniquely identified in FL and SL, respectively (Fig. 2a). However, this comparison was limited because the common SAGs in FL and SL show distinct temporal expression patterns during grain-filling. Thus, we categorized SAGs into two groups based on the similarities of temporal expression patterns of the 6365 SAGs between FL and SL (Methods): (1) 3047 genes that showed shared temporal expression patterns between FL and SL during grain-filling (Shared_SAGs, log_2_-fold-changes between FL and SL < 0.58 (1.5-fold) at all six time points), and (2) 3058 genes that showed differential temporal expression patterns between FL and SL during grain-filling (Diff_SAGs, log_2_-fold-changes ≥0.58 and P < 0.05 from t-test at one or more of the six time points) (Fig. 2b; Supplementary Table 1). Of the 6365 SAGs, 260 did not belong to Shared_SAGs or Diff_SAGs because, although they showed larger than 1.5-fold differences between FL and SL at one or more time points, the differences were not statistically significant (P > 0.05, t-test). These data show that FL and SL have shared and differential transcriptional programs during the grain-filling period.

### Cellular processes associated with Shared_SAGs in FL and SL during grain-filling

Several studies have shown that FL and SL perform similar roles to provide nutrients to grains^3, 33^, suggesting the presence of Shared_SAGs (3047 SAGs). To systematically explore the Shared_SAGs, we classified them into seven patterns (SP1-7) (Supplementary Fig. 1a) using k-means clustering followed by hierarchical clustering (Methods). The seven patterns of Shared_SAGs included four (SP1-4) up-regulated and three (SP5-7) down-regulated patterns (Supplementary Fig. 1a). To understand cellular processes associated with Shared_SAGs, we performed the enrichment analysis of gene ontology biological processes (GOBPs) for the genes in SP1-7 (Supplementary Fig. 1b and Table 2). First, the transport process was represented by SP1, which had a peaked pattern during grain-filling. SP1 included four major facilitator superfamily genes (*Os01g0268100*, *Os03g0795000*, *Os04g0573000*, and *Os12g0133100*), two ABC transporters (*Os08g0167000* and *Os04g0209200*), and a putative copper transporter (*Os08g0455900*), suggesting that mobilization of molecules using these transporters occurred commonly in both FL and SL during the grain-filling period. Second, DNA methylation/replication/repair, RNA processing, and production of ta-siRNAs/siRNAs involved in RNA interference were represented by SP2 and/or SP3 with early increases during grain-filling. Third, stress responses (responses to salt stress, cadmium ion, and phosphate starvation) were represented by SP4, with gradual increases during grain-filling. Finally, photosynthesis and Chl biosynthetic processes were represented by SP5 and/or SP6, which had early or gradual decreases during grain-filling. These data suggest that DNA repair, epigenetic gene silencing (DNA methylation and siRNA generation), and stress responses were up-regulated with shared temporal patterns in FL and SL during the grain-filling period, whereas photosynthesis-related processes were down-regulated with shared temporal patterns in FL and SL.

### Cellular processes associated with Diff_SAGs in FL and SL during grain-filling

**Figure 3:**
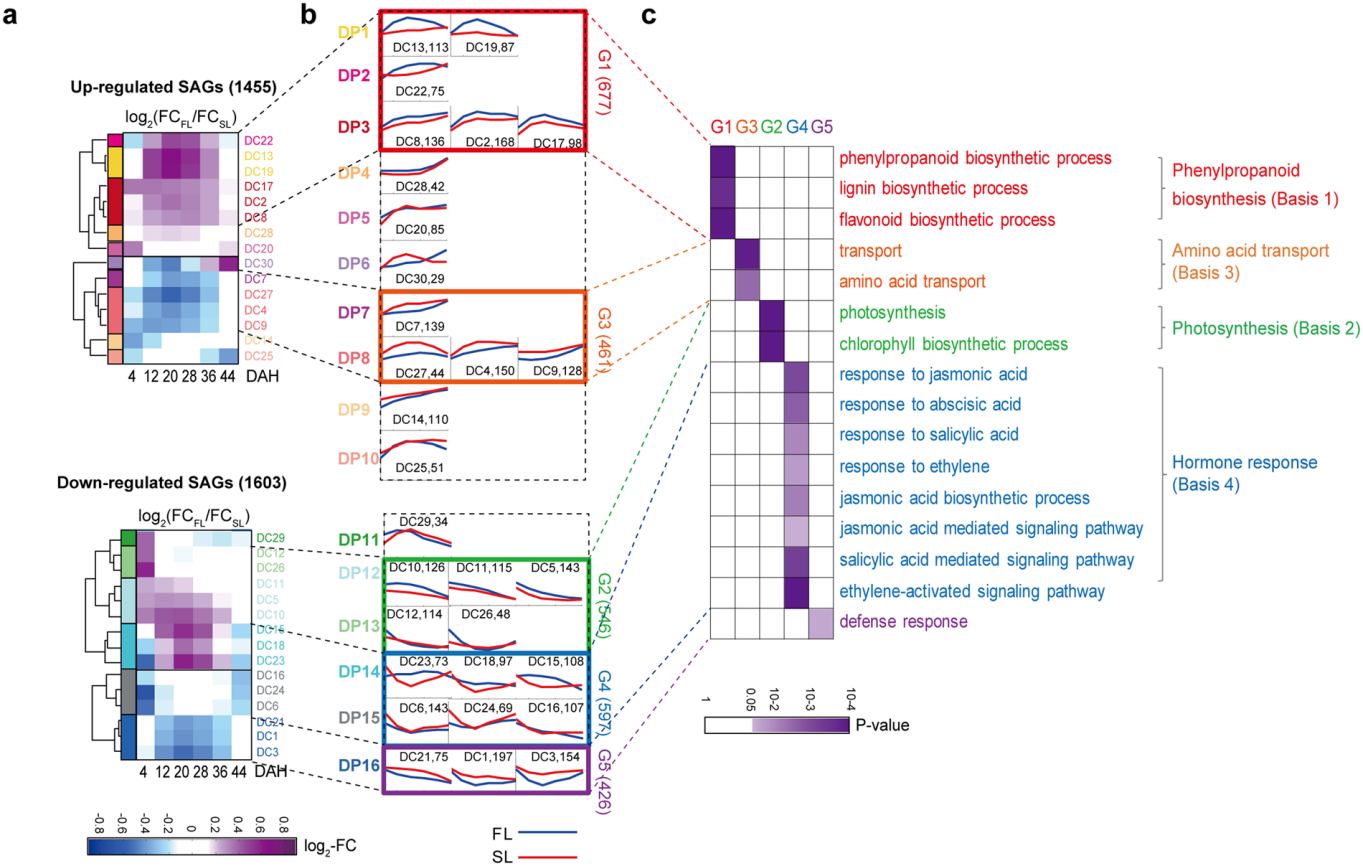
Differential SAGs between FL and SL. (**a**) Difference patterns of temporal gene expression profiles of Diff_SAGs between FL and SL. The 3058 Diff_SAGs were clustered into 30 clusters (DC1-30; Methods). The heat map shows mean differences of log_2_-FCs [log_2_(FC_FL_/FC_SL_)] values for genes in each cluster between FL and SL at each time point. Colors represent higher (purple) or lower (blue) log_2_-FCs values in FL than in SL. The color bar shows the gradient of log_2_-fold-change differences between FL and SL at each time point. DC1-30 were then grouped into 16 patterns (DP1-16) based on differential temporal expression patterns. Blue and red lines represent the average log_2_-FC profiles during grain-filling in FL and SL, respectively. The five major groups (G1-5) are denoted by colored boxes, relationships between G1-5 (log_2_-FC profiles) in (**b**) and DC1-30 [heat map in (**a**)] are indicated by dotted lines, and total numbers of genes in G1-5 are shown in parenthesis. (**c**) Gene ontology biological processes (GOBPs) significantly (P < 0.05) represented by Diff_SAGs in G1-5, and those enriched by G1-5 are highlighted in different colors (see Supplementary Fig. 2 and Table S3 for a detailed list of GOBPs). The color bar shows the gradient of log_10_(P) values, where P is the enrichment P-value from the EASE scoring method in DAVID software. Finally, representative GOBPs for G1-5 are presented.

Differential decreases of Chl levels and N contents in FL and SL during the grain-filling period (Fig. 1b–e) suggests the presence of Diff_SAGs in FL and SL during grain-filling. For the 3058 Diff_SAGs identified above, we initially computed differences between temporal mRNA expression profiles in FL and SL and classified them into 1455 up-regulated and 1603 down-regulated SAGs during senescence, based on temporal difference patterns (Fig. 3a). To systematically explore these Diff_SAGs, we then categorized them into 16 patterns (DP1–16) using the method used for Shared_SAGs (Methods). The 16 patterns of Diff_SAGs included 10 up-regulated (DP1-10) and six down-regulated (DP11–16) patterns (Fig. 3b). Of the DP1–16, we further focused on five major groups (G1-5) of the patterns that showed similar differences between temporal expression profiles in FL and SL (Fig. 3b), with more than 306 SAGs (>10% of the total number of Diff_SAGs).

To investigate cellular processes associated with Diff_SAGs, we performed the enrichment analysis of GOBPs for the genes in G1-5 (Fig. 3c; Supplementary Fig. 2 and Table 3). First, the synthesis of phenylpropanoid family compounds (flavonoid, phenylpropanoid, and lignin) was represented by G1 (Fig. 3c) and were up-regulated during grain-filling with higher levels in FL than in SL during the middle stage of grain-filling (DP1-3 in Fig. 3b). These three molecules are known to protect plants from various types of stresses^38–40^, and the pathways for their synthesis are largely shared with the common precursor phenylalanine. These data suggest that protection against stresses involving these molecules increases during grain-filling in both FL and SL, but that the protection capacity is higher in FL than in SL during the middle stage of grain-filling. Second, photosynthetic and Chl biosynthetic processes and lipid and carbohydrate metabolism were represented by G2 (Fig. 3c; Supplementary Fig. 2), which was down-regulated during grain-filling with higher levels in FL than in SL during the early stage of grain-filling (DP12-13 in Fig. 3b). These data suggest that C assimilation through photosynthetic activity decreases during grain-filling in both FL and SL, but is higher in FL than in SL during the early stage of grain-filling. Third, transport processes were represented by G3 (Fig. 3c), which was up-regulated during grain-filling with higher levels in SL than in FL during the middle stage of grain-filling (DP7-8 in Fig. 3b). G3 included transporters of various nutrients, such as nucleotides, sucrose, copper, phosphate, AAs, and peptides, and among these, AA transporters (AATs) were significantly (P < 0.05) enriched in G3 (Fig. 3c). These data suggest that N supply through AATs from both FL and SL to grains increases during grain-filling, but is higher in SL than in FL during the middle stage of grain-filling, as suggested by the data in Fig. 1d. Fourth, hormone responses (jasmonic acid, abscisic acid, and ethylene) associated with senescence were represented by G4 (Fig. 3c), which showed down-regulation during grain-filling and higher levels in SL than in FL during the early stage of grain-filling (DP14-15 in Fig. 3b). These data suggest that senescence-associated hormone responses decrease during grain-filling in both FL and SL, but are higher in SL than in FL during the early stage of grain-filling. Finally, defense responses were represented by G5 (Fig. 3c), which showed down-regulation during grain-filling with higher levels in SL than in FL during the middle stage of grain-filling (DP16 in Fig. 3b).

The GOBPs represented by Diff_SAGs can vary with the cutoffs used to select Diff_SAGs. To examine this aspect, we applied four more stringent cutoffs to select Diff_SAGs and found that the GOBPs for G1-4 were still represented by the Diff_SAGs that were identified using the stringent cutoffs, whereas defense responses for G5 were not (see Discussion). Thus, we focused on the aforementioned GOBPs represented by G1-4. These data suggest four potential molecular bases (Bases 1-4) for the differential mRNA expression patterns in FL and SL during the grain-filling period (Fig. 3c) as follows: (1) phenylpropanoid biosynthesis (Basis 1 for G1), (2) photosynthetic and Chl biosynthetic processes (Basis 2 for G2), (3) transport of AAs (Basis 3 for G3), and (4) senescence-associated hormone responses (Basis 4 for G4).

### Distinct associations of Shared_SAGs and Diff_SAGs with transport and photosynthesis

**Figure 4:**
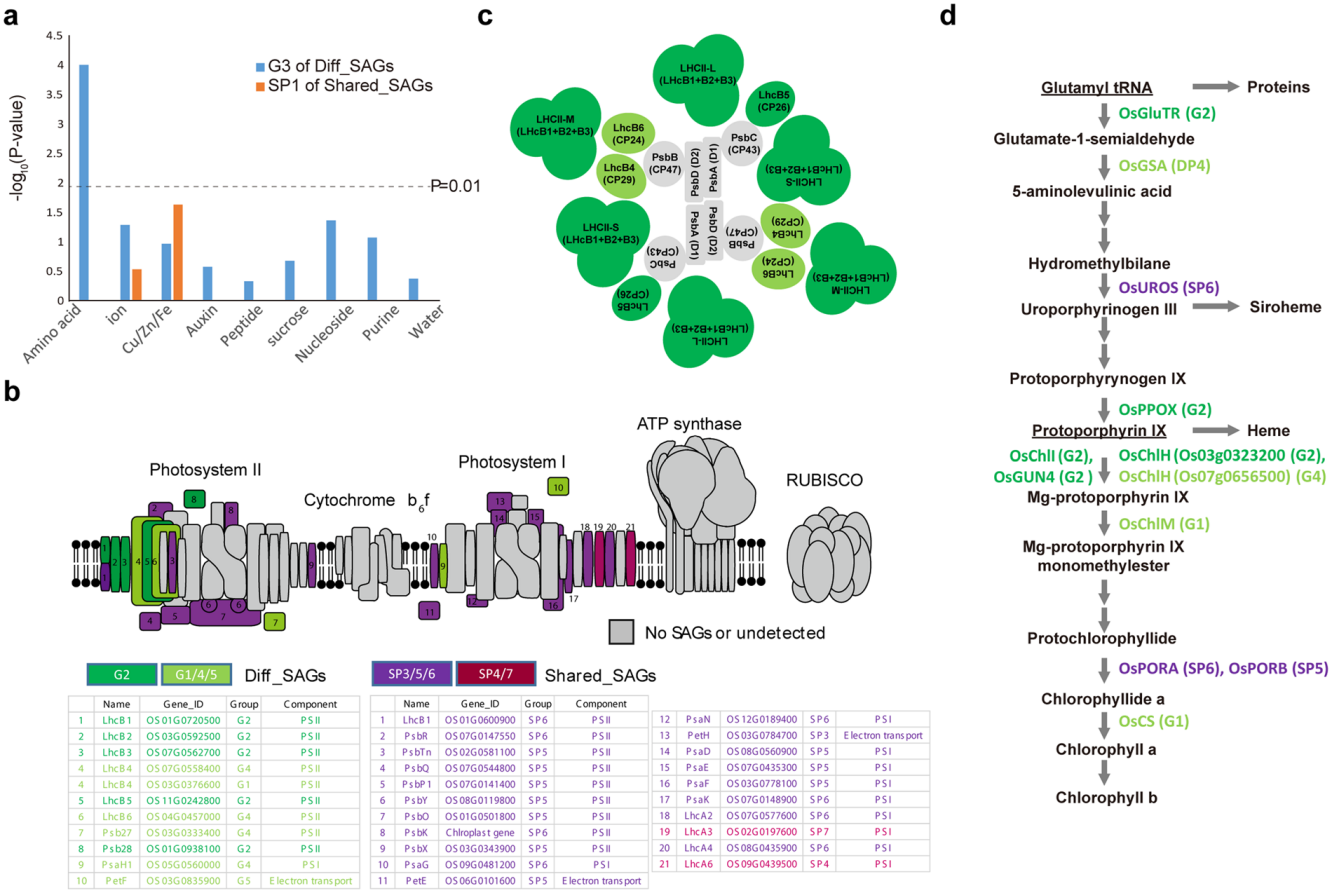
Distinct associations of shared and differential SAGs with transport, photosynthesis, and Chl biosynthesis. (**a**) Transporters included in G3 and SP1. The line represents the significance cutoff (P-value) for each type of transporter that was enriched by Diff_SAGs in G3 and Shared_SAGs in SP1. (**b**) Distributions of Diff_SAGs in G2 and Shared_SAGs in SP3 and SP5-6 across photosynthetic machinery (PS I and II, cytochrome b_6_f, ATP synthase, and RuBisCO complexes) on thylakoid membrane^96^; Diff_SAGs and Shared_SAGs are denoted in green and purple, respectively. Diff_SAGs in G1 and G4-5 and Shared_SAGs in SP4 and SP7 are denoted as light green and dark purple components, respectively. Gray components represent non-SAGs and show no significant expression changes during grain-filling. The table summarizes components with gene symbols, descriptions, and complexes to which they belong. (**c**) Top view of Diff_SAGs in PS II. The color scheme is used as in (**b**). (**d**) Metabolic pathway for Chl biosynthesis. Metabolites (e.g., Glutamyl tRNA) are denoted as nodes in the pathway, and enzymes for metabolic reactions, such as GluTR for glutamyl tRNA → glutamate-1-semaldehyde, in the pathway are denoted near the reaction arrows. Double arrows indicate omission of intermediate reactions. Patterns with corresponding enzymes are denoted in parenthesis. The color scheme for enzyme labels is used as in (**b**).

The above analyses show that several cellular processes are represented by both Shared_SAGs and Diff_SAGs as follows: (1) The transport process by SP1 of Shared_SAGs (Supplementary Fig. 1b) and by G3 of Diff_SAGs (Fig. 3c) and (2) photosynthesis and Chl biosynthetic processes by SP3, SP5, and SP6 of Shared_SAGs (Supplementary Fig. 1b) and also by G2 of Diff_SAGs (Fig. 3c). Shared_SAGs and Diff_SAGs included mutually exclusive sets of SAGs, suggesting that Shared_SAGs and Diff_SAGs were distinctly associated with different sets of transporters or with different subnetworks of photosynthesis and Chl biosynthesis. To confirm these distinct associations of Shared_SAGs and Diff_SAGs with transport processes, we examined the relative enrichment of subfamilies of transporters in SP1 of Shared_SAGs and G3 of Diff_SAGs. G3 of Diff_SAGs was significantly (P < 0.01, hypergeometric test) enriched with AATs (Fig. 4a; Supplementary Fig. 3 and Table 4). In contrast, SP1 of Shared_SAGs included various types of transporters, including three Cu/Fe/Zn, two ion, one phosphate, one folate, and one nucleotide, with no significant enrichment of particular types of transporters, suggesting that the supply of molecules through these transporters is common to FL and SL.

To confirm associations of Shared_SAGs and Diff_SAGs with photosynthesis, we mapped the genes in SP3 and SP5-6 of Shared_SAGs and in G2 of Diff_SAGs into the photosynthesis complexes: photosystem I (PS I), photosystem II (PS II), cytochrome b_6_f, ATPase, and RuBisCO (Fig. 4b). Among these, PS I and II were prominently enriched with the Shared_SAGs and/or Diff_SAGs. PS I was predominantly enriched with Shared_SAGs (8 SP5-6 SAGs), whereas PS II was enriched with both Shared_SAGs (9 SP5-6 SAGs) and Diff_SAGs (5 G2 SAGs). The five G2 Diff_SAG in PS II comprised the mobile light harvesting components (*OsLhcB1-3*)^41^, the adaptor component (*OsLhcB5*), and the assembly/repair component (*OsPsb28*)^42^ (Fig. 4b,c). In contrast, the nine SP5-6 Shared_SAG in PS II comprised lumen-exposed Psb components of oxygen-evolving complexes (*OsPsbO*/*P*/*Q*/*R*), which are conserved or specifically present in higher plants^43^ (Fig. 4b). However, no expression changes in the core components of PS II (*OsPsbB*/*D1*/*D2*) were observed during grain-filling (Fig. 4c). Collectively, these data suggest that PS I activity and PS II oxygen-evolving activity showed shared down-regulation patterns in FL and SL, whereas PS II light harvesting and assembly/repair activities showed differential down-regulation patterns in FL and SL.

Finally, we mapped G2 (Diff_SAGs) and SP5-6 (Shared_SAGs) into the Chl biosynthetic pathway (Fig. 4d). Five enzymes in the pathway, glutamyl-tRNA reductase (*OsGluTR*), protoporphyrinogen oxidase (*OsPPOX*), two subunits of magnesium-chelatase (*OsChlH*/*I*)^44^, and a proteinaceous cofactor genome-uncoupled 4 (*OsGUN4*)^45^, showed differential down-regulation patterns in FL and SL. These enzymes are key to the direction of metabolic flux into Chl biosynthesis at branching points in the metabolic pathway (Fig. 4d). In contrast, uroporphyrinogen III synthase (*OsUROS*) and protochlorophyllide reductases A and B (*OsPORA* and *OsPORB*), which catalyze several intermediate reactions in the pathway, showed shared down-regulation patterns in FL and SL. Collectively, these data suggest that Chl biosynthetic pathway activities showed largely differential down-regulation patterns in FL and SL, resulting in higher Chl levels in FL (Fig. 1b,c). However, the three intermediate enzymes in the pathway showed shared down-regulation patterns in FL and SL, likely contributing to the coordination of Chl biosynthesis in FL and SL.

### Effects of panicle removal on differential expression patterns of Diff_SAGs in FL and SL

**Figure 5:**
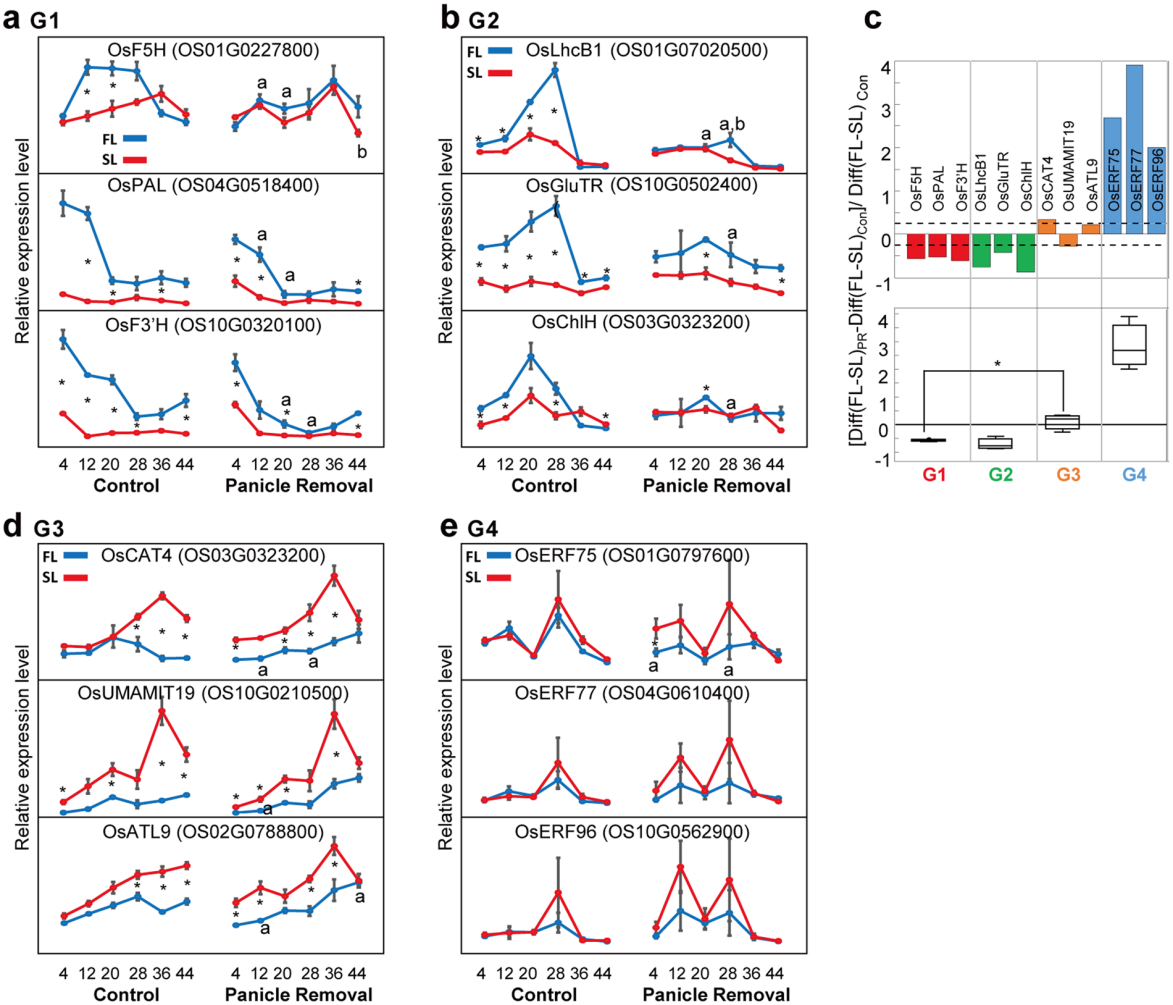
Effects of panicle removal on expression patterns of Diff_SAGs between FL and SL. (**a**,**b**,**d**,**e**) qRT-PCR analyses of independent samples (n = 3) performed for the denoted representative genes of G1-4 with and without panicle removal (“Panicle Removal” and “Control” panels, respectively). Expression levels of representative genes were normalized to those of actin at corresponding time points. Blue and red lines represent temporal expression profiles of genes in FL and SL, respectively. Data are presented as means ± SEM from three biological replicates. (**c**) Relative changes of integrated differences in temporal expression profiles of representative genes between FL and SL with and without panicle removal (top panel); [Diff(FL-SL)_PR_ − Diff(FL-SL)_Con_]/Diff(FL-SL)_Con_. Integrated differences with [Diff(FL-SL)_PR_] or without panicle removal [Diff(FL-SL)_Con_] were computed by integrating differences in expression levels at each time point using trapezoidal integration. Boxplots of relative changes in integrated differences for genes in each group are shown (bottom panel); *P < 0.05, two-tailed t-tests of denoted comparisons.

Grains serve as the major sink for nutrient remobilization from leaves, suggesting that grains may affect senescence of leaves during grain-filling. In agreement, Choudhuri *et al*.^28, 30^ previously showed that panicle removal changes senescence patterns of FL and SL from non-sequential to sequential. Thus, we investigated the relative effects of grain-filling on senescence of FL and SL in panicle removal experiments and measured temporal expression patterns of the following representative genes (Panicle Removal in Fig. 5a,b and d,e) for Bases 1–4 (G1–4 of Diff_SAGs; Fig. 3c): (1) ferulate 5-hydroxylase (*OsF5H*), phenylalanine ammonia-lyase (*OsPAL*), and flavonoid 3′-monooxygenase (*OsF3*′*H*) for phenylpropanoid biosynthesis (Basis 1 for G1); (2) light harvesting complex Chl a/b binding protein (*OsLhcB1*), glutamyl-tRNA reductase (*OsGluTR*), and magnesium-chelatase subunit H (*OsChlH*) for photosynthesis and Chl biosynthesis (Basis 2 for G2); (3) UMAMIT19 homolog (*OsUMAMIT19*), *OsATL9*, and *OsCAT4* for AA transport (Basis 3 for G3); and (4) *OsERF75*, *OsERF77*, and *OsERF96* for hormone responses (Basis 4 for G4). In addition to panicle removal experiments, we confirmed differential expression patterns of these representative Diff_SAGs in mRNA sequencing experiments (mRNA-seq panels in Supplementary Fig. 4a–d) using qRT-PCR analyses of independent cohorts of FL and SL grown under different field and environmental conditions (Control panels in Fig. 5a,b and d,e or Supplementary Fig. 4a–d).

The six representative genes for Bases 1 and 2 (G1 and 2 in Fig. 5a,b, respectively) were more highly expressed in FL than in SL, consistent to the observations from mRNA-sequencing (G1-2 in Supplementary Fig. 4a,b, respectively), thus supporting up-regulation of phenylpropanoid biosynthesis (Basis 1) and photosynthesis (Basis 2) in FL, compared with that in SL. Panicle removal reduced the differences in expression levels of all the six genes in FL and SL (Fig. 5c), mainly by decreasing expression levels in FL (Fig. 5a,b). Next, the six representative genes for Bases 3 and 4 (G3 and 4 in Fig. 5d,e, respectively) showed higher expression in SL than in FL, consistent to the observations from mRNA-sequencing (G3 and 4 in Supplementary Fig. 4c,d, respectively), thus supporting up-regulation of AA transport (Basis 3) and hormone responses (Basis 4) in SL, compared to that in FL. In contrast with Bases 1 and 2, the three representative genes for Basis 4 (G4 in Fig. 5e) tended to have increased expression differences in FL and SL following panicle removal (Fig. 5c), particularly at the early stages of grain-filling. However, panicle removal only had residual effects on differential expression of the three representative genes for Basis 3 (G3 in Fig. 5d) in FL and SL (Fig. 5c).

The qRT-PCR analyses show that relative expression levels of representative genes for Bases 1–4 in FL and SL are consistent with data from mRNA-sequencing analyses in cohorts grown under different conditions (Supplementary Fig. 4a–d). However, the kinetic patterns of up- or down-regulation of representative genes for Bases 1, 2, and 4 during grain-filling in mRNA-sequencing data were different from those in qRT-PCR data (Supplementary Fig. 4e). In contrast, mRNA sequencing and qRT-PCR analyses of differential expression patterns of the three representative genes for Basis 3 were significantly correlated (Supplementary Fig. 4e). Taken with panicle removal experiments, these data suggest that differential senescence-associated expression patterns for Basis 3 [AA transport (G3)] are invariant between cohorts, even after panicle removal, whereas the differential expression patterns for Bases 1, 2, and 4 [phenylpropanoid biosynthesis (G1), photosynthesis (G2), and hormone responses (G4)] differ significantly between cohorts and following panicle removal.

### Association of long-distance AATs with differential decreases of N contents between FL and SL

The above data suggest that Basis 3 (AA transport) may act as an invariant core differential senescence program between FL and SL, which should be robustly achieved under environmental variations during the grain-filling period. In contrast, the other bases likely serve as variable differential senescence programs between FL and SL. N is an essential nutrient for plant growth and seed development, and stable N supply to grains provides N for germination and seedling growth. Accordingly, N supply during the grain-filling period can be predominantly achieved by AA transport from leaves to grains.

**Figure 6:**
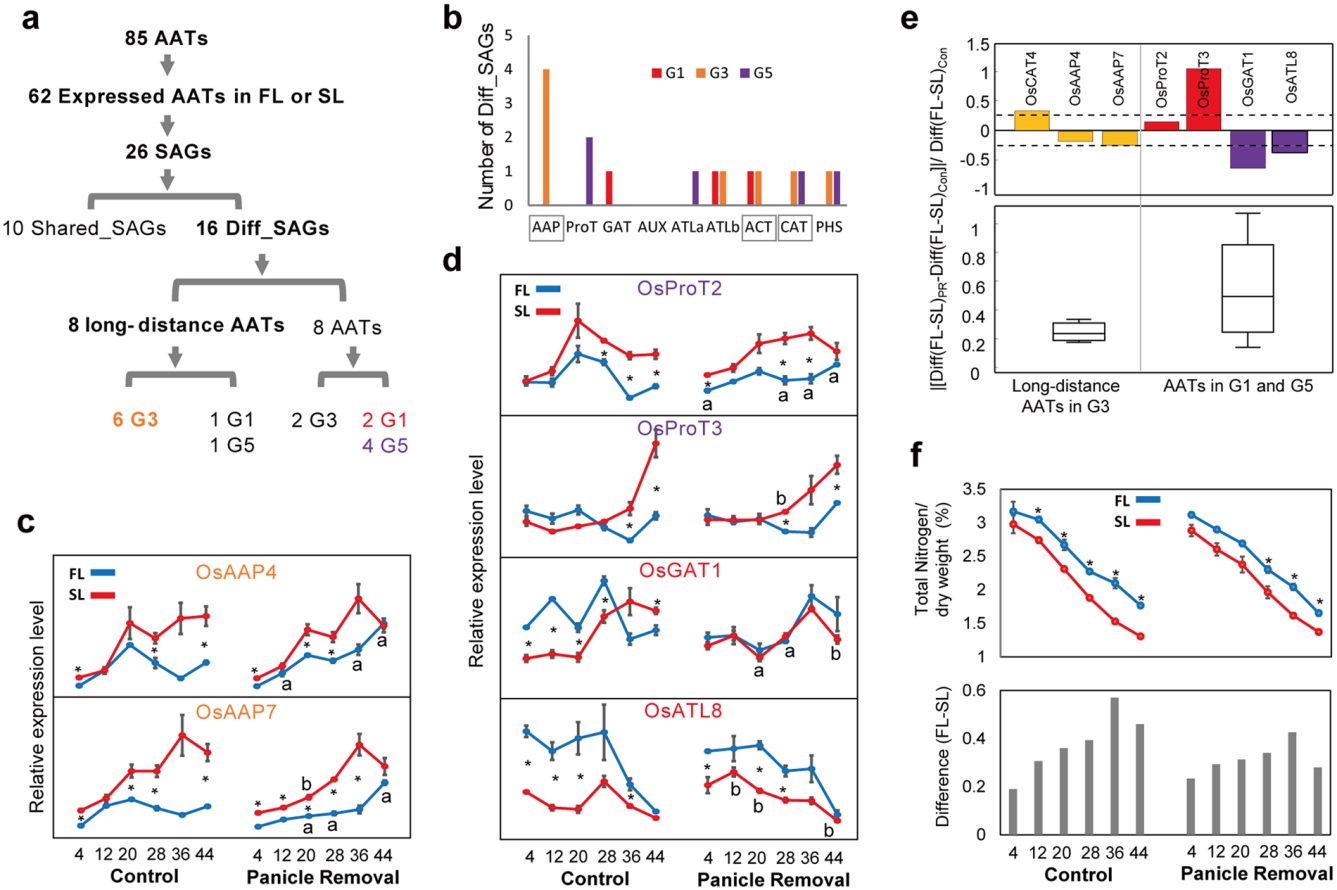
Long-distance AATs and differential N contents between FL and SL. (**a**) Selection procedures for long-distance AAT Diff_SAGs that reflect differential N content profiles between FL and SL (Fig. 1d). (**b**) Numbers of AAT Diff_SAGs (G1, G3, and G5) of individual AAT subfamilies (see also Supplementary Table 5). (**c**,**d**) qRT-PCR analyses of independent samples (n = 3) performed for denoted AAT genes with and without panicle removal (“Panicle Removal” and “Control” panels, respectively). Expression levels of AAT genes were normalized to those of actin at corresponding time points. Blue and red lines represent temporal expression profiles of genes in FL and SL, respectively. Data are presented as means ± SEM from three biological replicates. (**e**) Relative changes of integrated differences in temporal expression profiles of AAT genes between FL and SL with and without panicle removal (top panel); [Diff(FL-SL)_PR_ − Diff(FL-SL)_Con_]/Diff(FL-SL)_Con_. Integrated differences with [Diff(FL-SL)_PR_] or without panicle removal [Diff(FL-SL)_Con_] were computed by integrating differences in expression levels at each time point using trapezoidal integration. Boxplots for the absolute relative changes of integrated differences for the two groups of genes (three long-distance AAT genes and negative controls) are shown in the bottom panel. (**f**) Differential total N content profiles (g/dry weight %) during grain filling with and without panicle removal (“Panicle Removal” and “Control” panels, respectively; top panel). Blue and red lines represent temporal N content profiles in FL and SL, respectively. Differences in N contents between FL and SL are shown for each time point (bottom panel). *P < 0.05, two-tailed t-tests of denoted comparisons.

Thus, we compared the relevance of AATs to differential senescence programs in FL and SL and identified those that are associated with lower total N contents in SL than in FL (Fig. 1c). We first examined differential expression patterns of all AATs with senescence-associated expression changes in FL and SL. Among 85 AATs in the rice genome^46^, 62 were expressed in FL or SL during grain-filling (Fig. 6a), and of these, 26 were identified as SAGs of AAAP (17 AATs) and APC (9 AATs) AAT families. Finally, 16 were identified as Diff_SAGs (3, 8, and 5 in G1, G3, and G5, respectively) and 10 were identified as Shared_SAGs (4, 3, 2, and 1 in SP2, SP3, SP6, and SP7, respectively; Supplementary Table 5). Compared with the 10 AAT Shared_SAGs, the 16 AAT Diff_SAGs are likely more relevant to lower total N contents in SL than in FL (Fig. 1c). Thus, we examined subfamilies of the 16 AAT Diff_SAGs, because different AAT subfamilies transport different types of amino acids or other related metabolites. The 16 AAT Diff_SAGs belonged to the following subfamilies (Fig. 6b): AAP (4 AATs in G3), ProT (2 in G5), GAT (1 in G1), ATLa (1 in G5), ATLb (1 in G1 and 1 in G3), ACT (1 in G1 and 1 in G3), CAT (1 in G3 and 1 in G5), and PHS (1 in G3 and 1 in G5). Members of the AAP subfamily were previously identified as major AATs in long-distance transport of AAs with broad substrate specificity^37, 47^. Moreover, ACT and CAT subfamilies were predicted to be involved in the long-distance transport of AAs^37, 48, 49^. In contrast, ProT, GAT, and PHS subfamilies transport the small substrates proline/glycine/betaine/GABA, GABA, and polyamines, respectively^50–52^, whereas the transport substrates of ATLa/b remain unknown. Among 16 AAT Diff_SAGs, 8 AATs belonged to the three subfamilies of long-distance AA transporters, including 4 AAPs, 2 ACT, and 2 CAT, suggesting relevance to N supply from leaves to grains. Finally, 8 AATs were found in G1 (1 AAT), G3 (6 AATs), and G5 (1 AAT). G1 showed higher expression in FL than in SL, G5 showed down-regulation during grain-filling in SL, and G3 showed up-regulation during grain-filling. These data indicate increased AA transporting activities in SL, suggesting greater relevance to lower total N content in SL than in FL (Fig. 1c). Thus, of the eight long-distance AAT Diff_SAGs, the six AATs in G3 (4 AAPs, 1 ACT, and 1 CAT) can be more relevant to differential senescence programs between FL and SL.

Of the six long-distance AATs in G3, *OsCAT4* was invariant in its differential expression between FL and SL during grain-filling after panicle removal (Fig. 5d). Thus, we examined whether the other five long-distance AATs (*OsAAP4*/*5*/*7*/*8* and *OsBAT4*) were also invariant after panicle removal. We measured expression levels of these genes with and without panicle removal at the six time points during grain-filling. *OsAAP5*/*8* and *OsBAT4* were excluded from this analysis because expression levels of *OsAAP8* were too low for reliable measurement and in contrast with mRNA-sequencing data, *OsAAP5* and *OsBAT4* failed to show differential expression patterns between FL and SL in independent cohorts (Supplementary Fig. 5a). Thus, we examined panicle removal-invariant differential expression of the remaining two long-distance AATs *OsAAP4* and *OsAAP7* between FL and SL. These genes were more highly expressed in SL than in FL (Fig. 6c), as indicated by mRNA-sequencing analyses (Supplementary Fig. 5b), suggesting up-regulation of long-distance AA transport in SL. Furthermore, as observed in expression analyses of *OsCAT4*, panicle removal had only residual effects on the differences in expression levels of *OsAAP4* and *OsAAP7* between FL and SL (Fig. 6c and e). Subsequently, we measured expression levels of four AATs (*OsGAT1* and *OsATL8* in G1 and *OsProT2* and *OsProT3* in G5) that belonged to neither G3 nor the three long-distance AAT subfamilies (two of colored G1 and two of colored G5 AATs in Fig. 6a). Expression differences of *OsGAT1* and *OsATL8* between FL and SL were decreased by panicle removal (Fig. 6e, top panel), whereas those of *OsProT3* were increased (Fig. 6e, top panel). Thus, compared with these AATs, expression levels of the three long-distance AATs (*OsCAT4* and *OsAAP4*/*7*) in G3 were altered to a smaller degree in the differential expression patterns between FL and SL (Fig. 6e, bottom panel). Collectively, these data suggest that long-distance AATs are invariant in their differential expression patterns between FL and SL after panicle removal.

Long-distance AATs contribute to differences in N contents between FL and SL (Fig. 1d). Thus, we determined whether the differential N contents between FL and SL during grain-filling were also invariant regardless of the presence of the panicle. To this end, we compared differential N content profiles between FL and SL during grain-filling in independent cohorts with and without panicle removal. These comparisons showed that the differences in N contents between FL and SL during grain-filling were not significantly altered with and without panicle removal (Fig. 6f).

## Discussion

Leaf senescence significantly affects yield and quality of grains^4–7^. Previously, FL and SL in rice have been shown to have shared decreases in photosynthesis but different decreasing rates of Chl levels during grain-filling^28, 30, 31^. However, the molecular bases underlying shared and differential aging patterns have rarely been explored. In this study, we performed time-dependent transcriptome analysis of FL and SL during grain-filling and identified 6365 SAGs with aging-dependent expression changes in FL and/or SL. Subsequently, we categorized these into 3047 Shared_SAGs and 3058 Diff_SAGs according to temporal expression patterns in FL and SL. Shared_SAGs were mainly associated with DNA repair, epigenetic gene silencing (DNA methylation and siRNA generation), and stress responses, whereas Diff_SAGs were mainly associated with phenylpropanoid biosynthesis (Basis 1), PS II-based photosynthetic and Chl biosynthetic processes (Basis 2), transport of AAs (Basis 3), and senescence-associated hormone responses (Basis 4). These data indicated that Shared_SAGs and Diff_SAGs mediate shared and differential molecular aging patterns between FL and SL during grain-filling.

Leaf senescence increases N remobilization and decreases C assimilation. Because leaves are the major source of N and C for grains, it would be difficult to satisfy the needs of N and C simultaneously in grains during leaf senescence, referred to as the “dilemma of senescence”^53, 54^. According to our analyses of Bases 1–2 of the Diff_SAGs, FL showed higher expression levels of genes involved in photosynthesis (G2 in Fig. 3; light harvesting components, their adaptor molecules, and damage repair molecules of PS II in Fig. 4b) and in protection from ROS-generating environmental stresses (G1 in Fig. 3; flavonoid, phenylpropanoid, lignin synthesis-related genes)^38–40^. These data suggest higher capacities of light collection and recovery in PS II during photosynthesis and thus higher C assimilation in FL than in SL, as shown previously^30, 31, 55^. However, compared with FL, SL showed higher levels of genes involved in AA transport (G3 in Fig. 3; AATs in Fig. 4a) and had lower total N contents (Fig. 1d), suggesting higher N remobilization than in FL. This functional segregation of C assimilation and N remobilization to FL and SL, respectively, during grain-filling provides C and N to seeds and overcomes the dilemma of senescence. Although the dilemma of senescence was initially addressed in examinations of single leaves during the grain-filling period^53, 54^, our findings suggest that these issues may be resolved by examining multiple leaves simultaneously.

Chl and N levels decreased continuously during grain-filling, but expression levels of their associated genes, *OsSGR1*, *OsPPH1*, *OsRCCR1*, *OsNAP*, and *OsAAP5*, peaked prior to the end of grain-filling (Fig. 1c and e). In this study, we measured temporal expression patterns of these genes because previous studies have reported the association of these genes with Chl degradation (*OsSGR1* ^25, 26^, *OsPPH1* ^22^, and *OsRCCR1* ^24^) or N remobilization (*OsNAP* ^8^ and *OsAAP5* ^37^) during grain-filling. In contrast with previous studies that report expression levels of these genes up to 28 DAH^8^, we continued to measure their expression levels up to 44 DAH and showed peaks in temporal expression patterns at 28 DAH. In agreement with our data, the RiceXPro^56^ database includes the time-course gene expression profiles up to 35 DAH and shows that *OsSGR1*, *OsPPH1*, *OsRCCR1*, and *OsNAP* expression levels peak at 28 DAH (Supplementary Fig. 6). However, the mechanisms behind these peaks remain unclear, and potentially reflect unknown factors that prevent excessive decreases of Chl degradation and N remobilization at the late phase of grain filling.

In this study, we proposed four molecular bases (Bases 1–4) based on the GOBPs represented by the Diff_SAGs in G1–4 identified using the following criteria (Criteria 1): log_2_-fold-changes ≥0.58 and P < 0.05 from t-test at one or more of the six time points. However, the GOBPs for G1–4 (Bases 1–4) can vary depending on the cutoffs used to identify the Diff_SAGs. Thus, we applied the following four additional stringent criteria to identify Diff_SAGs: Criteria 2–3) log_2_-fold-changes ≥0.58 and P < 0.05 from t-test in at least two (Criteria 2) or three (Criteria 3) of the six time points; and Criteria 4–5) log_2_-fold-changes ≥1 and P < 0.05 in at least one (Criteria 4) or two (Criteria 5) of the six time points. Using Criteria 1–5, 3058, 1934, 1185, 960, and 518 Diff_SAGs were identified, respectively (Supplementary Fig. 7a), suggesting that log_2_-fold-changes are a more stringent cutoff for selection of Diff_SAGs than numbers of time points with expression differences between FL and SL. To examine the effects of these stringent cutoffs on the GOBPs represented by Diff_SAGs, GOBP enrichment analyses were performed for the Diff_SAGs identified using Criteria 2–5. The results showed that the GOBPs for G1–4 were still significantly (P < 0.05) represented by the Diff_SAGs identified using Criteria 1–4. In contrast, the GOBP (defense response) for G5 was not significantly represented by the Diff_SAGs identified using Criteria 2–5 while the GOBP (photosynthesis) for G2 was not significantly represented by the Diff_SAGs identified using the most stringent criteria (Criteria 5). These data suggest that defense responses for G5 are sensitive to differing cutoffs, but that the GOBPs for G1–4 are relatively robust. Moreover, the GOBPs for G1–4 can vary depending on the tool used to identify the Diff_SAGs. Thus, we applied the timecourse Package developed by Tei and Speed^57^ to our data and identified 2136 Diff_SAGs (P < 0.05; Methods). Among these, 969 (45.37%) overlapped with our Diff_SAGs, and 336, 180, 186, 248, and 132 belonged to G1-5, respectively. The GOBP enrichment analysis for these genes in G1-5 showed that the GOBPs for G1–4 were significantly represented by the corresponding Diff_SAGs (Supplementary Fig. 7b). Accordingly, we focused on the GOBPs for G1–4 (Bases 1–4), which are relatively robust against the criteria and the method used for identification of Diff_SAGs, and investigated differential expression patterns between FL and SL during grain-filling.

Diff_SAGs of Basis 3 representing AA transport showed invariant differential expression patterns between FL and SL after panicle removal (Fig. 5c,d) and between independent cohorts grown under different light and temperature conditions (Supplementary Fig. 4c,e). In contrast, Diff_SAGs of Bases 1, 2, and 4 showed variable differential expression patterns between FL and SL in independent cohorts and/or after panicle removal (Fig. 5). These three variable bases are consistent with findings from a number of previous studies. In particular, photosynthesis (Basis 2) is reportedly affected significantly by environmental factors, such as light, temperature, and states of nutrient sinks such as fruits and grains. Accordingly, (1) photosynthetic activity is inhibited under sub-optimal growth conditions involving non-optimal light or temperature^58–60^; and (2) photosynthetic activity is reduced when nutrient sinks are removed and the need for C assimilation decreases^61–65^. Similarly, the bases representing phenylpropanoid biosynthesis (Basis 1) and senescence-associated hormone responses (Basis 4) are reportedly sensitive to various environmental factors: (1) flavonoids can be induced by UV-B^66^; and (2) amounts of senescence-associated hormones, such as abscisic acid, ethylene, and jasmonic acid, can be varied in response to various types of stimuli^67^. Unlike the variable bases, however, no previous reports have shown that AA transport (Basis 3), particularly long-distance AA transport (Fig. 6), is invariant under different environmental conditions or in the absence of panicles (nutrient sinks).

Variable and invariant bases of differential expression patterns between FL and SL likely reflect frequently observed negative genetic relationships between grain yields and grain protein contents in crops, such as soybean, wheat, maize, and rice^68–71^. These negative relationships have been interpreted according to (1) competition between C and N for captured energy during grain development^72^ and (2) N dilution by C-based compounds^73^. Based on the present observations of variable and invariant bases, we suggest that the negative relationship between grain yield and protein content reflects differential sensitivity of photosynthesis (variable Basis 2) and AA transport (invariant Basis 3) to senescence-inducing stresses, such as drought, salinity, and diseases or pests^74^. Specifically, photosynthetic activity was sensitively down-regulated during stress-induced senescence, resulting in reduced grain yield. In contrast, AA transport activity was relatively stable, leading to stable protein contents in grains. Given the stable protein content, under stress conditions, photosynthetic activity can be further decreased, resulting in a yield relatively lower than can be explained by protein content in grains. Under non-stress conditions, however, photosynthetic activity can be less decreased, resulting in a yield relatively higher than indicated by protein contents.

Variable and invariant components are often observed in biological systems and in physical and chemical systems. These systems have a general structure wherein invariant components are located at the core and variable components are peripheral and interact with core components and external factors. For example, circadian clocks have the core clock components (LHY and TOC1), which are relatively stable under environmental variations. These core clock components constantly interact with variable components, including CCA1, ELFs, PRRs, GI, ZTL, and PIFs, at outer layers, which respond to changes in various environmental factors. Thus, the transcriptional programs for the regulation of differential aging in FL and SL likely form a similar structure to ensure the stability of core components, such as stable protein contents in grains through differential long-distance AA transport for N remobilization from SL, and to cope with environmental changes through variable bases (phenylpropanoid biosynthesis, photosynthesis, and hormone defense responses. These hypotheses are supported by observations that (1) photosynthesis and plant growth are down-regulated until favorable conditions are recovered, and (2) when the need for C in newly developing sinks increases, photosynthesis increases in source leaves. Furthermore, analyses of other systems indicate close interactions of the core basis (AA transport) with variable bases.

Finally, our findings can be useful in developing transgenic plants and farming practices (e.g., fertilizers) that optimize rice grain yields and protein contents under given conditions. Our results suggest that leaves ensure first the limiting N resource in grains through differential long-distance transport of AAs between FL and SL regardless of environmental conditions. Moreover, invariant supplies of AAs to grains likely maintain minimal plant growth when photosynthesis is severely blocked, because AAs can be used as the C source. For example, glutamate dehydrogenase produces the TCA cycle intermediate 2-oxoglutarate from glutamate^75–78^. Hence, invariant supplies of AAs can also facilitate seed germination even in N limiting soil. Taken together, these data suggest that the stable supply of AAs to grains is of high priority and is met by differential aging regulation between FL and SL. Concomitantly, variable bases accommodate environmental variations. Therefore, transgenic rice or farming practices that enhance long-distance AA transport in SL can be developed to maximize the benefits of differential aging regulation between FL and SL.

## Methods

### Plant materials and sampling

Rice seedlings of the Asian rice cultivar Dongjin (*Oryza sativa L*. *ssp*. *Japonica*) for mRNA-sequencing were transplanted in a paddy field at Daegu, South Korea (approximately 35 °N) in early June in 2013. Seedlings for qRT-PCR analyses (control and panicle removal) were transplanted in a different field at the same time in 2014. In both years, heading dates were around August 20, and stems with similar heading dates were marked for sample preparation and physiological analyses. Samples were prepared between 7 and 8 AM at six time points (4, 12, 20, 28, 36, and 44 days after heading). Middle parts^16^ of nine independent leaves were obtained as a biological replicate and three replicates were sampled at the same time.

### Library construction and mRNA-sequencing

Rice leaf tissues were ground using a mortar and pestle, and total RNA was extracted from tissue samples of approximately 100 mg using RNeasy Plant Mini Kit (Qiagen). RNA integrity was confirmed using an Agilent Technologies 2100 BioAnalyzer (RNA integrity number >8.0). Poly(A) mRNA was then isolated from total RNA, and fragmentation reactions were performed using Illumina Truseq Stranded mRNA LT Sample Prep Kit with poly-T oligo-attached magnetic beads according to the manufacturer’s instructions. Reverse transcription of RNA fragments was performed using Superscript II reverse transcriptase (Life Technologies, Grand Island, USA). Adaptor-ligated libraries were generated for the resulting samples and were then sequenced using Illumina HiSeq 2500. Paired-end sequencing was performed to 101 bp from both ends for three independent replicates at each time point under individual conditions.

### Alignment and quantification of sequenced reads to the *Oryza sativa* genome

For the read sequences obtained from mRNA-sequencing, adapter trimming was performed using cutadapt software^79^ (version 1.2.1) with the adapter sequences (TruSeq Universal Adapter and indexed TruSeq^TM^ Adapters, provided by Illumina). Read alignment was performed using TopHat aligner^80^ (version 2; -r 100–library-type fr-firststrand–mate-std-dev 100–solexa-quals -p 24) with two mismatches and up to 10 multiple alignments allowed based on *Oryza sativa* reference genome (*Oryza sativa* IRGSP-1.0.22). We then assembled the aligned reads into transcripts based on gene features (GTF file of *Oryza sativa* IRGSP-1.0.22. gtf) and used Cufflinks^81^ (version 2.0.2;–library-type fr-firststrand -N–max-bundle-frags 1000000000 -F 0–min-frags-per-transfrag 1–max-bundle-length 5500000) to calculate the fragments per kilobase per million mapped reads (FPKM) values for the assembled transcripts. Quantitated transcripts whose FPKMs are larger than 1 in at least one time point were regarded as expressed transcripts as previously described^82^. For the statistical test and pattern analysis of the transcripts, we obtained the aligned read counts using HTSeq.^83^ (version 0.6.1) based on gene features and then performed a two-stage normalization as previously described^84, 85^. Briefly, we first normalized the aligned read counts into count per million (CPM) using The trimmed mean of M-values (TMM) normalization method^86^ in edgeR package^87^ to remove the bias of RNA composition and the difference of library sizes across the samples and then performed the quantile normalization^88^ on log_2_(CPM + 1) to remove systematic variation in the CPM values across the samples. Note that one was added to CPM before taking the log to avoid negative infinity.

### Identification of SAGs

The normalized log_2_-CPM values were divided into time-course expression profiles of FL and SL. The time-course expression profiles of FL or SL were smoothed using the Savitzky-Golay method^89^ with polynomial order = 2 and frame size = 5. For each transcript, we calculated the Pearson correlation coefficients (PCCs) between the smoothed expression profiles from three possible pairwise combinations of the three biological replicates (replicate 1 vs. 2, 2 vs. 3, and 3 vs. 1). The transcripts with the median PCCs ≥0.7 were selected as the ones with reproducible age-dependent expression patterns. The cutoff of 0.7 was determined from the following random experiments: (1) for *n* × *m* time-course mRNA expression data matrix (X) for *n* transcripts and *m* time points in each replicate of FL or SL, we randomly permuted the columns of X; (2) for each transcript, we calculated PCC between temporal expression profiles using the randomly permuted data matrixes (Xrand) of the replicates; (3) steps 1 and 2 were repeated 100 times; and (4) we estimated an empirical distribution for the null hypothesis (a transcript showed no correlation between the temporal expression profiles) using the PCCs obtained from the random permutations. The 95^th^ percentile of PCC in the empirical null distribution was 0.71. Based on this finding, we determined the PCC cutoff to be 0.7. The distribution of the number of transcripts with respect to PCC with an interval of 0.7 from zero to one revealed that for FL, there were 17696 (46.5%) with significant (P < 0.05) correlations (PCC ≥ 0.7), 4079 (10.7%) with marginal correlations (0.4 ≤ PCC ≤ 0.7), and 8945 (23.5%) with low correlations (PCC < 0.4), and for SL, there were 13681 (36.0%) with significant (P < 0.05) correlations with (PCC ≥ 0.7), 5671 (14.9%) with marginal correlations (0.4 ≤ PCC ≤ 0.7), and 10956 (28.8%) with low correlations (PCC < 0.4). For FL, we selected the transcripts with the median PCC ≥ 0.7 based on the time-course expression profiles of FL and then identified FL_SAGs as the selected transcripts with P < 0.05 (ANOVA with Bonferroni correction as a post-hoc test) and the maximum absolute log_2_-fold-changes ≥1 over six time points. Using the same approach, we selected the transcripts with the median PCCs ≥0.7 based on the time-course expression profiles of SL, and then identified SL_SAGs. Finally, we defined overall SAGs as the union of FL_SAGs and SL_SAGs. For the overall SAGs, log_2_-fold-changes were calculated by subtracting the average log_2_-CPM values at 4 DAH in FL from those at individual time points. These log_2_-fold-changes for FL and SL were displayed in Fig. 2b (heat map in red and green colors).

### Identification of Shared_SAGs and Diff_SAGs

For each of the aforementioned overall SAGs, log_2_-fold-changes between FL and SL were calculated by subtracting the median log_2_-CPM values of the three replicates of SL from those of FL at individual time points. Of the overall SAGs, Shared_SAGs were identified as the SAGs with log_2_-fold-changes <0.58 (1.5-fold) for all six time points. We also applied the two-sample t-test to log_2_-CPM values of the three replicates of FL and SL at each time point to evaluate the significance (P-value) of the difference in expression levels between FL and SL at the corresponding time point. Of the overall SAGs, Diff_SAGs were then identified as the SAGs with P-values < 0.05 from the t-test and absolute log_2_-fold-changes ≥0.58 (1.5-fold) in one or more of the six time points. Moreover, to examine whether the method used to identify Diff_SAGs can affects the GOBPs represented by Diff_SAGs, we also identified Diff_SAGs by applying the timecourse Package developed by Tei and Speed^57^ to our data. For each gene, the Package provides no P-values, but only Hotelling T^2^ statistic values representing the significance of the difference between its temporal expression profiles of FL and SL. Thus, to estimate *P*-values for the Hotelling T^2^ values, we performed the following random permutation experiments: (1) for *n* *×* *m* time-course mRNA expression data matrix (X) for *n* genes and *m* time points in each replicate of FL or SL, we randomly permuted the columns of X; (2) for each gene, we calculated a Hotelling T^2^ value by applying the timecourse Package to the randomly permuted data matrixes (Xrand) of the replicates of FL and SL; (3) steps 1 and 2 were repeated 1000 times; (4) we estimated an empirical distribution for the null hypothesis (a gene showed no difference in the temporal expression profiles of FL and SL) using Hotelling T^2^ values obtained from the random permutations; and (5) for the Hotelling T^2^ value of each gene, we calculated an adjusted P-value by one-sided test using the empirical distribution. We then selected 2136 Diff_SAGs with P ≤ 0.05 and of these, 336, 180, 186, 248, and 132 belonged to G1-5, respectively.

### Pattern analysis of Shared SAGs and Diff SAGs

Shared_SAGs were clustered using their log_2_-fold-change profiles by the k-means clustering method with correlation as a similarity measure and the number of clusters (k) = 30. The 30 clusters (SC1-30) of Shared_SAGs were categorized into 16 up-regulated and 14 down-regulated clusters. The up- and down-regulated clusters were further grouped into SP1–4 and SP5-7, respectively, by applying hierarchical clustering (complete linkage method and Euclidean dissimilarity measure) to the average log_2_-fold-change profiles of the up- and down-regulated clusters. Also, Diff_SAGs were clustered using their log_2_-fold-change profiles and also additionally the differences of log_2_-fold-changes between FL and SL by the same k-means clustering method. The 30 clusters (DC1-30) of Diff_SAGs were then categorized into 15 up-regulated and 15 down-regulated clusters. The up- and down-regulated clusters were grouped into DP1-10 and DP11–16, respectively, by applying the same hierarchical clustering used for the Shared SAGs. Finally, DP1–16 were further categorized into five major groups (G1-5) based on the similarity in the difference patterns of log_2_-fold-changes between FL and SL.

### Enrichment analysis of GOBPs

GOBP annotations of *Oryza sativa* were collected from gramene (release 41)^90^, NSF45K annotation^91^, and RGAP all GO slim^92^. GOBPs enriched by a group of the genes in SP1-7 and G1-5 were identified using curated GOBP annotations by the EASE scoring method used by DAVID software^93^. For each group, we selected the GOBPs with P-values ≤ 0.05 and the numbers of genes with the GOBPs ≥5.

### qRT-PCR analyses

After DNase (AM1906, ambion) treatment, cDNA was synthesized from approximately 400 ng of total RNA using an ImProm-II Reverse Transcription System (A3800, Promega). Subsequently, qRT-PCR analyses were performed using CFX96 or CFX384 Touch™ Real-Time PCR Detection Systems (Bio-Rad) with cDNA from 10–15 ng of total RNA. Actin mRNA expression was used as an internal standard, and normalized cycle threshold (Ct) values were used in statistical analyses (Supplementary Table 6). All primer sequences are listed in Supplementary Table 6.

### Comparison of differences between expression profiles of FL and SL with and without panicle removal

Mean relative expression levels of three replicates in FL and SL measured from qRT-PCR analysis were first computed at individual time points, and the differences between the mean relative expression levels of FL and SL were then calculated at individual time points. The integrated difference with [Diff(FL-SL)_PR_] or without panicle removal [Diff(FL-SL)_Con_] was then calculated by integrating the differences of the mean expression levels at individual time points over time using the trapezoidal integration method. [Diff(FL-SL)_PR_ − Diff(FL-SL)_Con_]/Diff(FL-SL)_Con_ were calculated. The same method was used for long-distance AATs and the negative control AATs.

### Correlation between mRNA-sequencing and qRT-PCR data

For each of the representative genes for G1-5, log_2_-CPM values from mRNA-sequencing and the expression levels from qRT-PCR in FL or SL were smoothed using the Savitzky-Golay method with polynomial order = 2 and frame size = 5 as previously described^82^. The correlation coefficients between the smoothed mRNA-sequencing and qRT-PCR data were calculated using three different methods (Pearson, Spearman and Kendall’s methods). In each method, the correlation coefficients for FL and SL were weighted summed. For mRNA-sequencing or qRT-PCR data, the weights for FL and SL were calculated as the areas under the smoothed expression profiles of FL and SL, respectively. For FL or SL, the final weight was then computed as the geometric mean of the weights of the corresponding leaf for mRNA-sequencing and qRT-PCR data. The weighted summed correlation coefficients from the three methods were finally combined into their geometric mean.

### Analyses of chlorophyll and total N content

Chls were extracted from approximately 50 mg of ground tissue samples using 95% ethanol. Chl contents were calculated after measuring optical densities using a 96 well plate reader (Infinite M200 PRO, TECAN)^94^. Ground leaf tissues were then dried and total N contents were measured from approximately 2 mg tissue samples using an Elemental analyzer (Elementar).

### Data Availability

The mRNA sequencing data were deposited into the Gene Expression Omnibus^95^ with the accession number GSE89233.

## Electronic supplementary material

Supplementary information

Supplementary dataset
