# Supplementary material for: Molecular bases for differential aging programs between flag and second leaves during grain-filling in rice
Source: Sci Rep. 2017 Aug 18;7:8792. doi: 10.1038/s41598-017-07035-9 (PMC5562787; doi:10.1038/s41598-017-07035-9)
Supplement: Supplementary file 1 — Supplementary information [file 41598_2017_7035_MOESM1_ESM.pdf]

Molecular bases for differential aging programs between flag and second leaves during grain-filling in rice

Shinyoung Lee[1,#], Hyobin Jeong[1,#], Sichul Lee[1], Jinwon Lee[1], Sun-Ji Kim[1], Ji-Won Park[1], Hye Ryun Woo[2], Pyung Ok Lim[2],Gynheung An[3], Hong Gil Nam [1,2,*], and Daehee Hwang[1,2,*]

[1]Center for Plant Ageing Research, IBS, Daegu Gyeongbuk Institute of Science and Technology, Daegu 711-873, Republic of Korea; [2]Department of New Biology, Daegu Gyeongbuk Institute of Science and Technology, Daegu 711-873, Republic of Korea; and [3]Department of Plant Molecular Systems Biotechnology and Crop Biotech Institute, Kyung Hee University, Yongin 446-701, Republic of Korea.

This file includes:

Supplementary Methods

Supplementary Figures 1-7

Supplementary Tables 1-6

– Tables 1-4 are provided in additional Excel spreadsheets.

Supplementary References

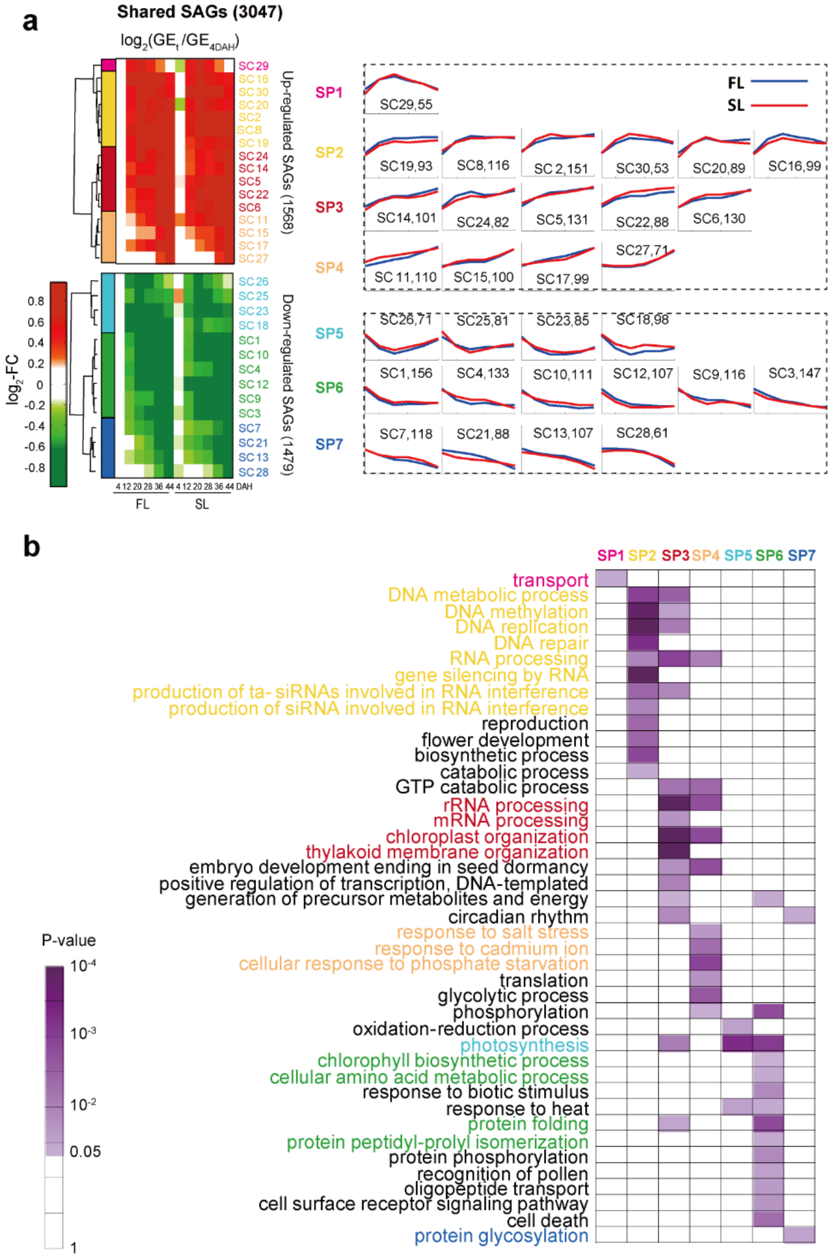

Supplementary Fig. 1. Shared senescence-associated genes (SAGs) between flag leaves (FL) and second leaves (SL). (**a**) Shared temporal gene expression (GE) patterns of Shared_SAGs between FL and SL. The 3,047 Shared_SAGs were clustered into 30 clusters (SC1-30; Methods). For each cluster, the heat map shows mean $\log_2$-FCs [$\log_2(GE_t / GE_{4DAH})$] of the resident genes at each time point (t), compared with that at 4 DAH. Colors represent up-regulation (red; $\log_2$-FC ≥ 0) and down-regulation (green; $\log_2$-FC < 0) during grain-filling, compared with that at 4 DAH in FL and SL. SC1-30 were then grouped into seven patterns (SP1-7) based on similarities of temporal expression patterns in individual clusters. Blue and red lines represent mean $\log_2$-FC profiles during grain filling in FL and SL, respectively. (**b**) Gene ontology biological processes (GOBPs) that were significantly ($P < 0.05$) represented by the Shared_SAGs in SP1-7. Key GOBPs enriched by SP1-7 are highlighted in different colors. The color bar shows gradient of $\log_{10}(P)$ values, where P is the enrichment P-value obtained from DAVID software.

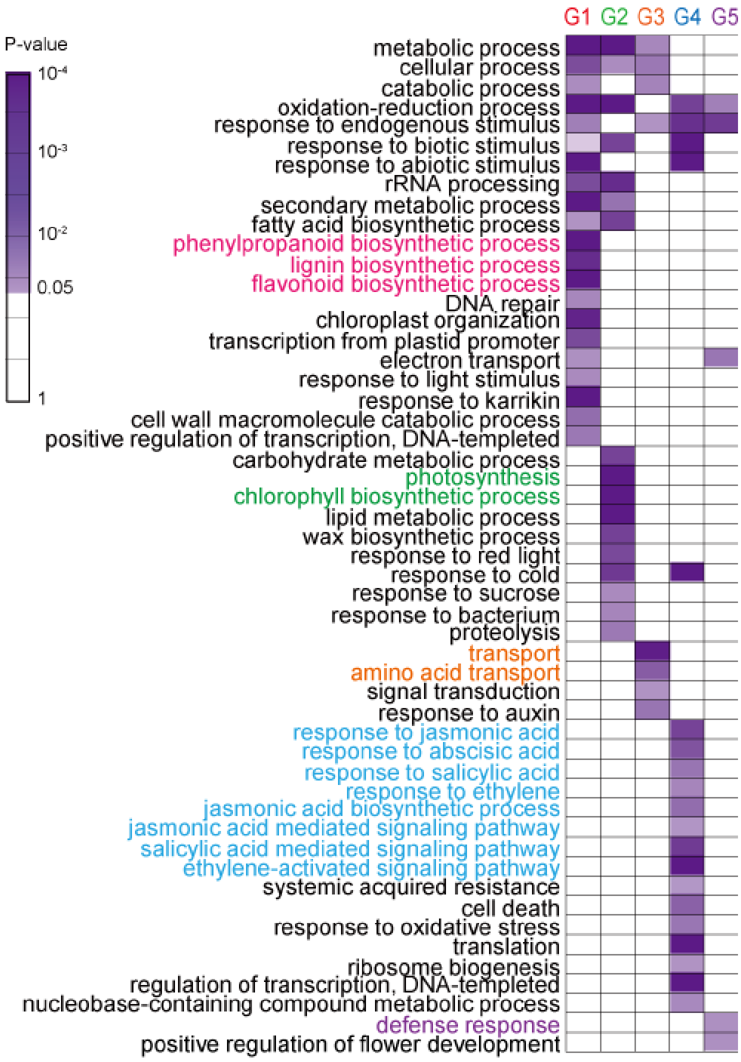

Supplementary Fig. 2. GOBPs represented by Diff_SAGs in G1-5. Key GOBPs that were enriched by G1-5 are highlighted in different colors. The color bar shows the gradient of $\log_{10}(P)$ values, where P is the enrichment P-value from DAVID software.

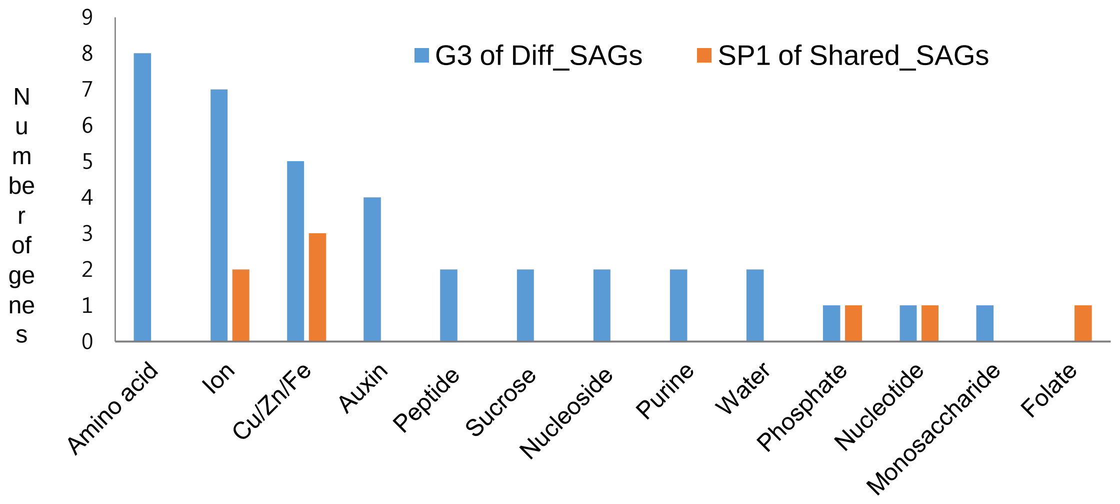

Supplementary Fig. 3. Numbers of transporters in G3 and SP1. A total of 13 types of transporters were included in G3 and SP1. Numbers of transporter types in G3 (blue) and SP1 (orange) are shown.

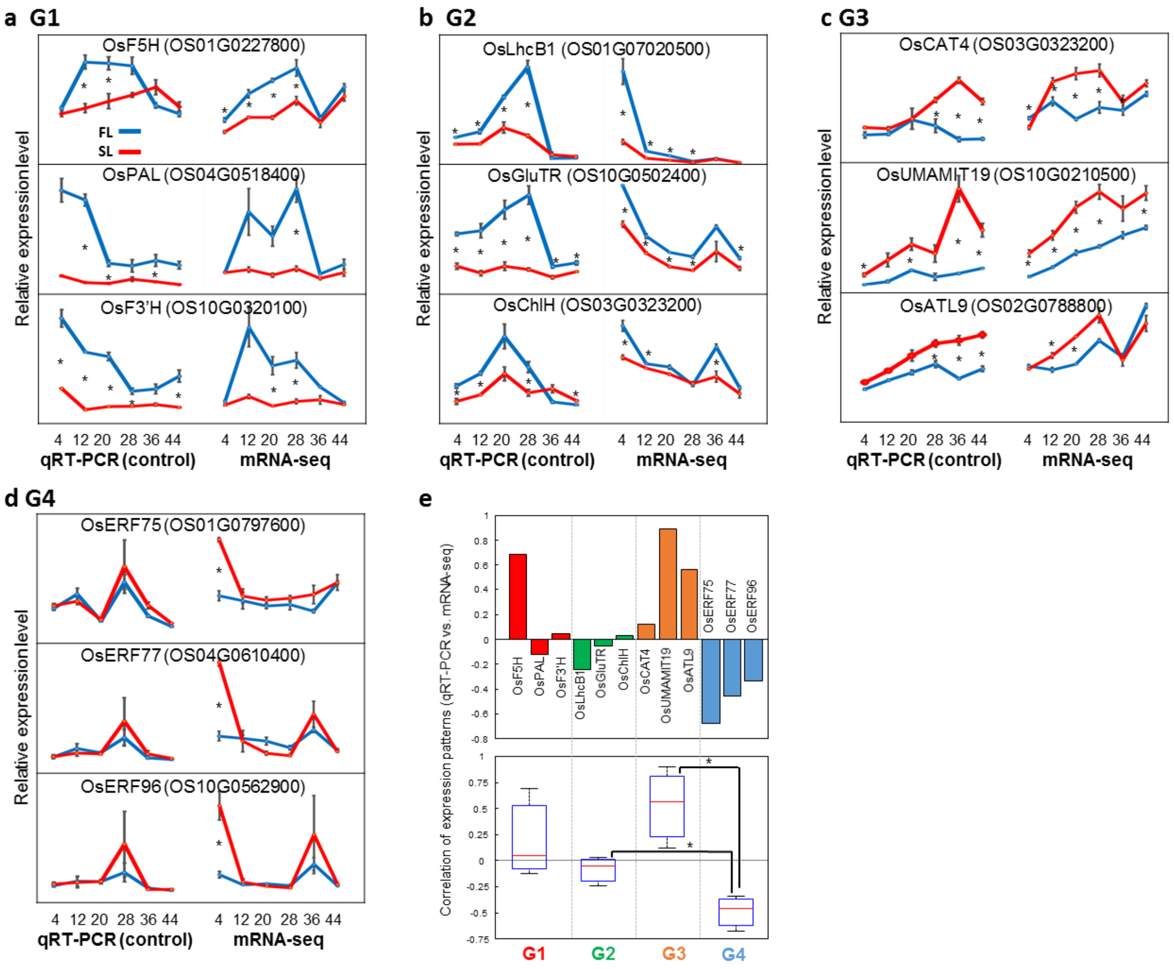

Supplementary Fig. 4. Comparison of temporal expression profiles in FL and SL from mRNA-sequencing and qRT-PCR analyses of independent samples. (**a-e**) qRT-PCR analyses of independent samples (n = 3) were performed for denoted representative genes of G1-4 (**a-d**, "qRT-PCR" panel, respectively). Expression levels of representative genes were normalized to those of actin at the corresponding time point. Blue and red lines represent temporal gene expression profiles in FL and SL, respectively. Read counts of representative genes from mRNA-sequencing are presented in the same manner (**a-d**, "mRNA-seq" panel). Data are presented as means ± SEM from three biological replicates. (**e**) Correlations of differential expression patterns of representative genes between FL and SL (top panel). Boxplots of gene correlations in each group are shown (bottom panel). *P < 0.05, two-tailed t-test for the denoted comparisons.

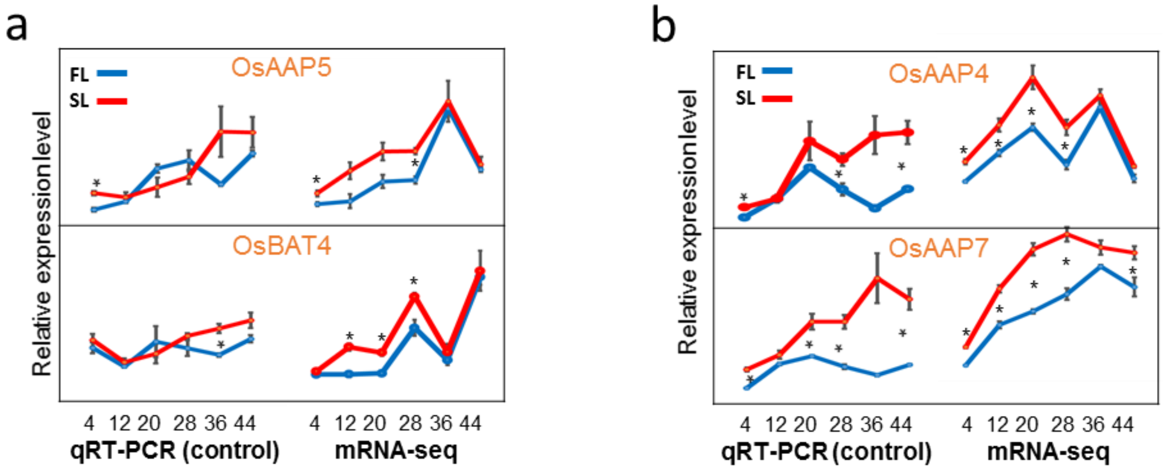

Supplementary Fig. 5. Comparison of temporal expression profiles in FL and SL from mRNA-sequencing and qRT-PCR analyses of independent samples. (**a-b**) qRT-PCR analyses of independent samples (n = 3) performed for the denoted AAT genes ("qRT-PCR" panel). Expression levels of AAT genes were normalized to those of actin at corresponding time points. Blue and red lines represent temporal gene expression profiles in FL and SL, respectively. Read counts of the AAT genes from mRNA-sequencing analyses are presented in the same manner (**a-b**, "mRNA-seq" panel). Data are presented as means ± SEM from three biological replicates. *P < 0.05, two-tailed t-tests for denoted comparisons.

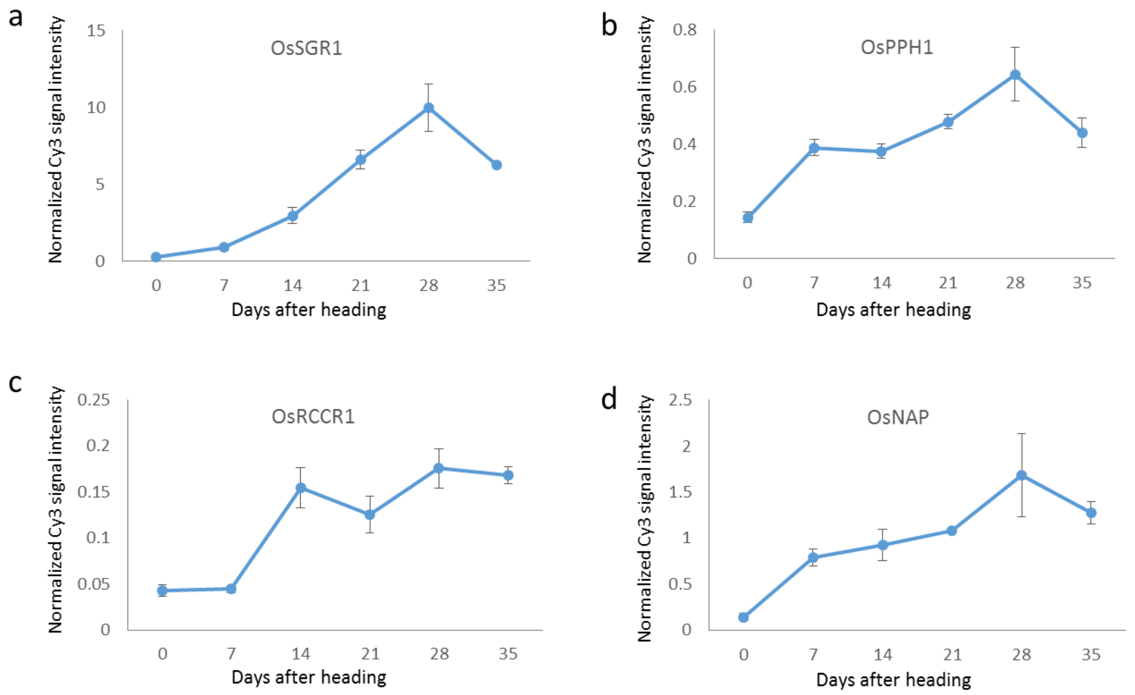

Supplementary Fig. 6. Temporal expression patterns of genes that are associated with decreased Chl and N contents during grain-filling. (**a-d**) Temporal mRNA expression profiles of *OsSGR1* (**a**), *OsPPH1* (**b**), and *OsRCCR1* (**c**) associated with Chl degradation, and those of *OsNAP* (**d**) associated with N remobilization according to the RiceXpro database; mRNA expression profiles were determined in FL at 0, 7, 14, 21, 28, and 35 DAH.

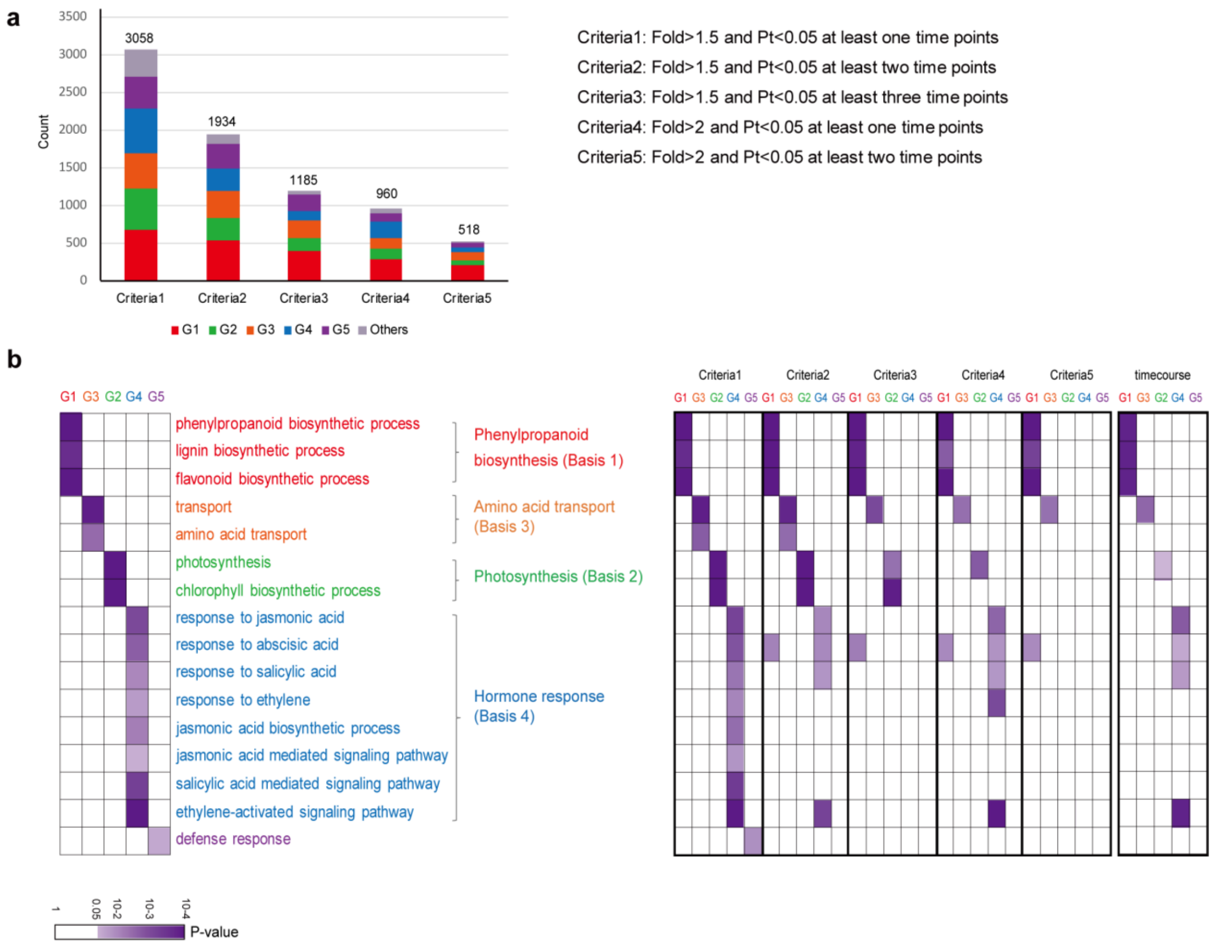

Supplementary Fig. 7. Diff_SAGs identified using various criteria and their associated GOBPs. (**a**) Numbers of Diff_SAGs that were identified using the following five criteria: Criteria 1) $\log_2$-fold-changes ≥ 0.58 and P-value (P_t) < 0.05 from t-test at one or more of the six time points; Criteria 2) $\log_2$-fold-changes ≥ 0.58 and P-value < 0.05 at two or more of the six time points; Criteria 3) $\log_2$-fold-changes ≥ 0.58 and P-value < 0.05 at three or more of the six time points; Criteria 4) $\log_2$-fold-changes ≥ 1 and P-value < 0.05 at one or more of the six time points; and Criteria 5) $\log_2$-fold-changes ≥ 1 and P-value < 0.05 at two or more of the six time points. For each of the criteria, the stacked bar graph shows numbers of Diff_SAGs in the five major groups (G1-5). Total numbers of Diff_SAGs identified using these criteria are shown above the stacked bar graph. (**b**) The heat map showing P-values of GOBPs that are enriched by the Diff_SAGs identified by Criteria 1-5 in each group. The color bar indicates the gradient of $-\log_{10}(P)$, where P is the enrichment P-value from the EASE method employed in DAVID software. The timecourse Package was used to identify Diff_SAGs and their enriched GOBPs using David software (see Discussion). The final heat map shows P-values of GOBPs that were enriched by the Diff_SAGs identified by the timecourse Package.

Supplementary Table 1. List of 6365 senescence-associated genes (SAGs). Gene IDs and descriptions for the SAGs are shown with log_2-normalized read counts and log_2-fold-changes (log2-FC) at the six time points (DAH 4, 12, 20, 28, 36, and 44). FL and SL columns show whether a gene was identified as a SAG ("SAG") or not (blank) in FL and SL, respectively. The "Shared/Diff_SAGs" column indicates whether a gene was classified as Shared_SAG or Diff_SAG.

See "Table S1" spreadsheet in the excel file entitled "Supplementary Tables.xlsx"

Supplementary Table 2. GOBPs represented by Shared_SAGs in SP1-7. GOBP term ID, term description, numbers of genes in SP1-7 with the GOBP (Count), and enrichment P-values (P-value) are shown in the table. Highlighted GOBPs in red (Enrichment) with P-values < 0.05 and counts ≥ 5 were considered GOBPs that were enriched by Shared_SAGs in SP1-7. Highlighted GOBPs in green are shown in Supplementary Fig. 1b.

See "Table S2" spreadsheet in the excel file entitled "Supplementary Tables.xlsx"

Supplementary Table 3. GOBPs represented by Diff_SAGs in G1-5 GOBP term ID, term description, numbers of genes in G1-5 with the GOBP (Count), and enrichment P-values (P-value) are shown in the table. Highlighted GOBPs in red (Enrichment) with P-value < 0.05 and counts ≥ 5 were considered GOBPs that were enriched by Diff_SAGs in G1-5. Highlighted GOBPs in green are shown in Fig. 3c.

See "Table S3" spreadsheet in the excel file entitled "Supplementary Tables.xlsx"

Supplementary Table 4. Transporters included in G3 and SP1. Gene ID, TIGR gene ID, the type of molecules transported (Category), and group or pattern to which each transporter belongs (Group) are shown in the table. Reference information for categories of transported molecules were obtained from the TAIR database and are presented in the table with detailed information from "Function", GOBP, gene ontology molecular function (GOMF), and cellular component (GOCC) in the TAIR database.

See "Table S4" spreadsheet in the excel file entitled "Supplementary Tables.xlsx"

Supplementary Table 5. List of 26 AAT SAGs Gene ID, TIGR gene ID, gene symbol, and group, or pattern to which it belongs (Group) are shown with AAT family and subfamily for each AAT.

Family	Subfamily	TIGR_ID	Gene_ID	Symbol	Group
AAAP	AAP	LOC_Os07g04180	OS07G0134000	OsAAP1	SP6
		LOC_Os12g09300	OS12G0194900	OsAAP4	G3
		LOC_Os01g65660	OS01G0878400	OsAAP5	G3
		LOC_Os01g65670	OS01G0878700	OsAAP6	SP3
		LOC_Os05g34980	OS05G0424000	OsAAP7	G3
		LOC_Os01g66010	OS01G0882800	OsAAP8	G3
		LOC_Os04g39489	OS04G0470700	OsAAP13	SP7
		LOC_Os12g08090	OS12G0181500	OsAAP16	SP6
	ProT	LOC_Os03g44230	OS03G0644400	OsProT2	G5
		LOC_Os07g01090	OS07G0100800	OsProT3	G5
	GAT	LOC_Os05g50920	OS05G0586500	OsGAT1	G1
	AUX	LOC_Os01g63770	OS01G0856500	OsAUX1	SP2
		LOC_Os05g37470	OS05G0447200	OsAUX2	SP2
	ATLa	LOC_Os02g09810	OS02G0191300	OsATL6	G5
	ATLb	LOC_Os11g19240	OS11G0298000	OsATL8	G1
		LOC_Os02g54730	OS02G0788800	OsATL9	G3
		LOC_Os04g38680	OS04G0460300	OsATL13	SP2
APC	ACT	LOC_Os01g42234	OS01G0607200	OsBAT1	SP3
		LOC_Os01g71720	OS01G0945300	OsBAT4	G3
		LOC_Os01g71740	OS01G0945600	OsBAT5	SP2
		LOC_Os01g71760	OS01G0945700	OsBAT6	G1
	CAT	LOC_Os03g43970	OS03G0641200	OsCAT3	SP3
		LOC_Os03g45170	OS03G0654400	OsCAT4	G3
		LOC_Os12g42850	OS12G0623500	OsCAT11	G5
	PHS	LOC_Os02g47210	OS02G0700500	OsLAT1	G5
		LOC_Os01g19850	OS01G0304100	OsLAT8	G3

Supplementary Table 6. List of primer sequences used for qRT-PCR gGene ID, TIGR gene ID, gene symbol, and forward and reverse primer sequences are shown for each gene.

	GeneID	TIGR_Gene_ID	Forward Primer	Reverse Primer
OsSGR1	Os09g0532000	LOC_Os09g36200	CGCATGCAATGTCGCCAAATG	GCTCACCACACTCATTCCCTAAAG
OsPPH1	Os06g0354700	LOC_Os06g24730	ATCAAGGGAGCTGGAATTTGT	CTTCAAAAGCGAACGAAGTTG
OsRCCR1	Os10g0389200	LOC_Os10g25030	GGATCCATGCTCCAACTCCGCTCG	AAGCTTCTAGGCCTCTTGTACCCCA
OsNAP	Os03g0327800	LOC_Os03g21060	TTGGTGCAACTTTCCAAATAGG	ATTCGCCATGTGCAATTATGTT
OsAAP5	Os01G0878400	LOC_Os01g65660	CAGGTCGGAACTTGGAACAT	CAAAGGCCGTTGTACAGGTT
OsF5H	OS01G0227800	LOC_Os01g12770	ACCGGAGAAATTCCTCAACAGCAC	CAAATGGTAGCAGCTCGAAGTCC
OsPAL	OS04G0518400	LOC_Os04g43800	GGGTCATGAACAGCATGATGAACG	CCGGCATTAAGAAACCGGATAAGC
OsF3'H	OS10G0320100	LOC_Os10g17260	AACGACCTTCTAAGCGTGCTG	CCGCAGTGAATAGGTTCAGGAG
OsLhcb1	OS01G0720500	LOC_Os01g52240	TCTCCATGTTCGGGTTCTTC	CCGGAGATCCACACTCACTT
OsGluTR	OS10G0502400	LOC_Os10g35840	GGACTCGCTGGAGACTGTTC	CGATCTTCTGGAGGCACTTC
OsChlH	OS03G0323200	LOC_Os03g20700	TACTACTTCCGCGCAACACTCC	CCATGCTTCAGCAACAAGCG
OsCAT4	OS03G0654400	LOC_Os03g45170	TTCTTCGCCGATGTTAACAA	CAGAGCTGCTGCACAGAGAC
OsUMAMIT19	OS10G0210500	LOC_Os10g14920	TGAGCTACCATTGTCCACGACTAATG	TGCCTGAAGCTTTCGTTGTCGATG
OsATL9	OS02G0788800	LOC_Os02g54730	TGACACAGACGGTGTTCAATGGG	TGGAGTGGAAAGAAGTCCAACACC
OsERF75	OS01G0797600	LOC_Os01g58420	CGTTCGCTTTCCTTTCAGAG	TCGGAGTCACTTTGTGCAAC
OsERF77	OS04G0610400	LOC_Os04g52090	CCCACAAATCCCTCGTAGAA	TGCACGGGGAGATAGGTATG
OsERF96	OS10G0562900	LOC_Os10g41330	TATTGCTGCTCAGGTGATCG	TTGCTCTGAGCCTCTGATGTT
OsAAP4	OS12G0194900	LOC_Os12g09300	GCAACATCGCCTTCTCCTAC	AAGACGGTGGTCATGGAGAG
OsAAP5	OS01G0878400	LOC_Os01g65660	CAGGTCGGAACTTGGAACAT	CAAAGGCCGTTGTACAGGTT
OsAAP7	OS05G0424000	LOC_Os05g34980	GTTCAGGCTGACATGGAGGT	CATCTCCACCGGGAAGTAGA
OsBAT4	OS01G0945300	LOC_Os01g71720	CTTGGATCACTGGCTGGTT	GACGTAGTTGGAGGCCATGT
OsProT2	OS03G0644400	LOC_Os03g44230	GTGGTCAAGAATATGGAGAA	ATAGCTTGATGTTGAGGACC
OsProT3	OS07G0100800	LOC_Os07g01090	AGGCCAGTCTACCTGCAAGA	CCCCAGTTGTCAGAACGAAT
OsGAT1	OS05G0586500	LOC_Os05g50920	CAATGCGTTCCTCTCCATT	AACAACACCACCGTGTAGCA
OsATL8	OS11G0298000	LOC_Os11g19240	ATGGTCGGAATGCTCATAGG	CCGATGTCCTGGTACGTCTT
OsACT1	OS03G0718100	LOC_Os03g50885	GTATCCATGAGACTACATACAACT	TACTCAGCCTTGGCAATCCACA
